# Cell-free placental DNA: What do we really know?

**DOI:** 10.1371/journal.pgen.1011484

**Published:** 2024-12-09

**Authors:** Natalie Yuen, Melanie Lemaire, Samantha L. Wilson

**Affiliations:** 1 Department of Biochemistry and Biomedical Sciences, McMaster University, Hamilton, Ontario, Canada; 2 Department of Obstetrics and Gynecology, McMaster University, Hamilton, Ontario, Canada; Stanford University School of Medicine, UNITED STATES OF AMERICA

## Abstract

Cell-free placental DNA (cfpDNA) is present in maternal circulation during gestation. CfpDNA carries great potential as a research and clinical tool as it provides a means to investigate the placental (epi)genome across gestation, which previously required invasive placenta sampling procedures. CfpDNA has been widely implemented in the clinical setting for noninvasive prenatal testing (NIPT). Despite this, the basic biology of cfpDNA remains poorly understood, limiting the research and clinical utility of cfpDNA. This review will examine the current knowledge of cfpDNA, including origins and molecular characteristics, highlight gaps in knowledge, and discuss future research directions.

## Author summary

Cell-free DNA are fragments of free-floating DNA released by cells into blood circulation. During pregnancy, the placenta releases cell-free placental DNA (cfpDNA) into maternal blood circulation. CfpDNA can be collected through a routine blood draw and is used for noninvasive prenatal testing (NIPT) to detect risk of the fetus having genetic abnormalities. There remains great untapped potential for the use of cfpDNA to screen not only for a wider range of fetal genetic abnormalities, but also for pregnancy complications affecting the health of both the fetus and pregnant person. This is currently hindered by a poor understanding of the “normal” characteristics of cfpDNA and how exactly it is released from placenta cells. In this review, we will summarize what is currently known about cfpDNA, highlight what remains unclear, and discuss future research directions for advancing the utility of cfpDNA in both research and the clinic.

## Introduction

**Fig 1:**
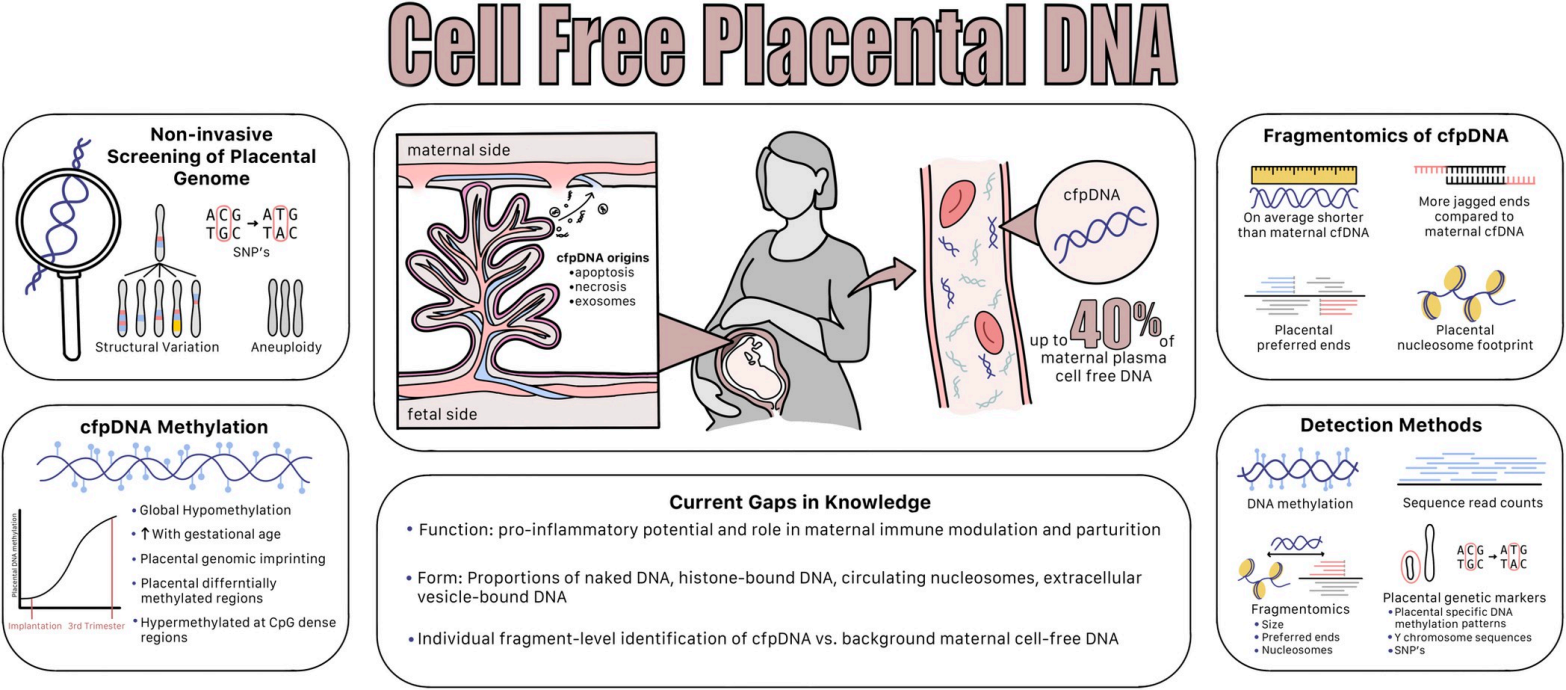
The current knowledge surrounding cfpDNA.

Cell-free placental DNA (cfpDNA) is shed into maternal circulation throughout pregnancy [1]. CfpDNA has been detected as early as the fourth week of gestation and has been estimated to comprise up to approximately 40% of total maternal plasma cell-free DNA [2–4]. Since its discovery in 1997, cfpDNA has been used in the clinical setting for noninvasive prenatal testing (NIPT) to screen for fetal genetic aberrations, most notably chromosomal aneuploidies. However, the understanding of the basic biology underlying cfpDNA remains limited, placing constraints on the information that can be obtained from analyzing cfpDNA. Gaps in knowledge include cfpDNA fragmentation processes, release mechanisms, form, and function. CfpDNA carries the potential for real-time study and monitoring of placental health and development. Further characterization of physical, genetic, and epigenetic characteristics of cfpDNA will pave the way for further basic research and clinical applications. In this review, we will examine the current understanding of cfpDNA, highlight gaps in knowledge, and discuss future research directions (Fig 1). While this review focuses on pregnancy, much of what is discussed applies to the cell-free DNA field in general, including other clinical contexts such as cancer.

## Placental development, anatomy, and dysfunction

**Fig 2:**
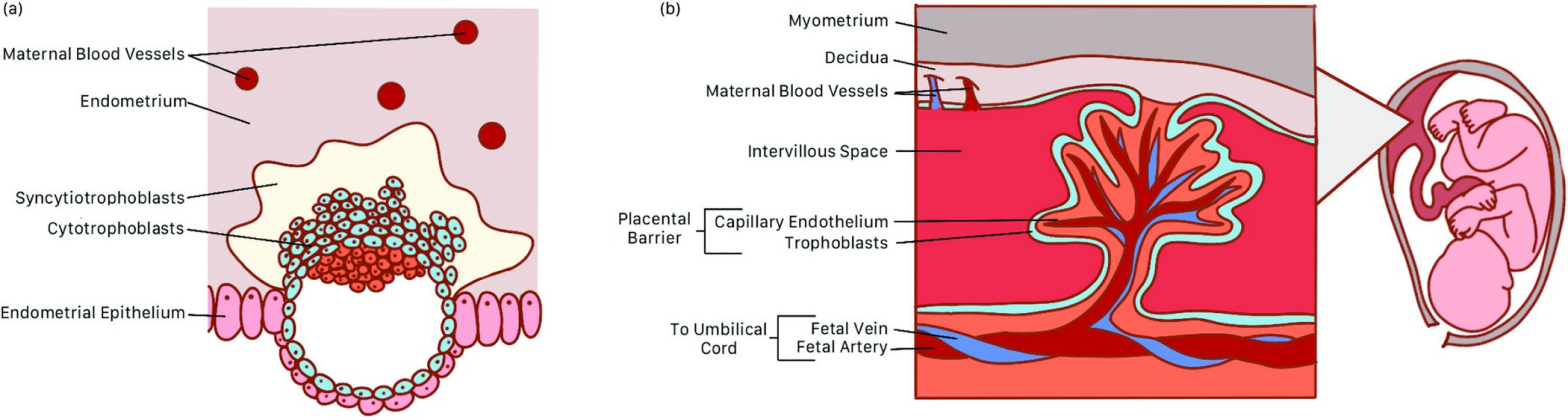
Placental development and anatomy. (A) Early invasion of the endometrium by trophoblasts during blastocyst implantation. (B) Chorionic villus and surrounding structures at the fetal–maternal interface.

The placenta is a transient organ that forms during gestation. It facilitates the exchange of molecules between the fetal and maternal bloodstreams to support the physiological needs of the developing feto-placental unit. The placenta arises from the trophectoderm layer of the blastocyst, which further differentiates into cytotrophoblasts, syncytiotrophoblasts, and extravillous trophoblasts [5]. Trophoblasts invade and reconstruct the innermost layer of the uterus, the endometrium (Fig 2A). Within the decidua, the differentiated and reconstructed layer of the endometrium, the invading trophoblasts develop into tree-like projections called chorionic villi (Fig 2B). Chorionic villi are composed of an inner stromal core and outer trophoblast layer [6]. Mesenchymal cells in the stromal core form capillaries which circulate fetal blood from the umbilical blood vessels [7]. The spaces between chorionic villi form the intervillous space which is eventually filled with maternal blood. Extravillous trophoblasts invade and remodel uterine spiral arteries, veins, and glands in the endometrium to establish maternal blood circulation and glandular secretion in the intervillous space [8]. Maternal blood circulation to the intervillous space is initially blocked by trophoblast plugs in the uterine vessels. At around 10 to 12 weeks of gestation, trophoblast plugs are displaced as spiral artery remodeling progresses and maternal blood flow to the intervillous space is initiated [9]. The layers of the chorionic villi, namely the trophoblasts and fetal capillary endothelium, form the placental barrier, which maintains separation of the fetal and maternal bloodstreams [6]. At the placental barrier, molecules including nutrients, gases, and hormones are exchanged between fetal and maternal blood. Various mechanisms are used by molecules to cross the placental barrier including passive and facilitated diffusion, active transport, and vesicular transport [10]. Through this exchange, oxygen and nutrients are supplied to the fetus while waste products, such as carbon dioxide, exit the fetal bloodstream to be excreted by the mother. The placenta also carries out an endocrine function by secreting hormones to signal the maternal body to maintain an environment that supports feto-placental growth throughout gestation [11].

Abnormal placental morphology and function is observed in various pregnancy complications. Preeclampsia is the new onset of maternal hypertension and proteinuria after 20 weeks of gestation which can lead to multi-organ system failure [12]. The condition is heterogeneous in the range and severity of symptoms and placental lesions. Therefore, there is not one specific cause of preeclampsia, rather multiple disease subtypes with different etiologies that are currently being elucidated [13–16]. Shallow trophoblast invasion, improper spiral artery remodeling, and the resulting poor placental perfusion has been a long-standing model for the etiology of preeclampsia [17]. It is believed that this causes placental hypoxia which leads to a systemic maternal response and endothelial dysfunction [18]. Other factors including maternal cardiovascular health are also believed to contribute to preeclampsia pathogenesis [17,18]. Preeclampsia poses serious short- and long-term risks to both the pregnant person and offspring including fetal growth restriction, cardiovascular disease later in life, and mortality [19]. Fetal growth restriction is characterized by pathologically poor fetal growth [20]. Among other factors, it can be caused by inadequate oxygen and nutrient transport to the fetus due to abnormal placentation and poor placental perfusion [21]. Fetal growth restriction impacts the development of the fetus’ organs and body systems, increasing risks of morbidities across the lifespan [22]. Therefore, proper placental development and function are critical for maternal and offspring health both during and after pregnancy.

The etiologies of many pregnancy complications remain poorly understood. Work over the past several years has revealed the roles of various molecular networks in placental development and function in both healthy and complicated pregnancies [23–25]. Advancements in understanding the molecular basis of pregnancy complications are invaluable for the creation of treatments and screening and diagnosis tools for earlier and more targeted intervention. Further research utilizing new technologies and models, such as cfpDNA, will help to drive these necessary advancements.

## CfpDNA origins

Despite commonly being referred to as “cell-free fetal DNA,” there is a large body of evidence demonstrating that “cell-free fetal DNA” mainly originates from trophoblasts, the major cell type of the placenta. Therefore, it is more accurate to refer to “cell-free fetal DNA (cffDNA)” as “cell-free placental DNA (cfpDNA).” Reports of normal first trimester cfpDNA levels in anembryonic pregnancies [26], the presence of cfpDNA before the establishment of fetal circulation [27], cell-free DNA release in in vitro placental explant studies [28–32], and cases of confined placental mosaicism in which cfpDNA corresponds to trophoblast karyotypes [33–40] provide direct evidence supporting the trophoblastic origin of cfpDNA. Additionally, detection of cell-free DNA carrying placenta-specific DNA methylation patterns in maternal plasma [41–44] and correlation between the placental and cfpDNA methylomes [45, 46] solidify the placenta as the main source of cfpDNA. Reports of increased cfpDNA concentrations in conditions involving placental dysfunction, such as preeclampsia [47–49], invasive placenta (placenta accreta and increta), and placenta previa [50] point towards the placental origin of cfpDNA as these observations are hypothesized to be due to increased trophoblast cell death. In this review, we will use the term “cffDNA” when referring specifically to DNA originating from the fetus and use “cfpDNA” when referring to DNA originating from the placenta. The term “cell-free DNA” will be used to describe cell-free DNA in general, regardless of tissue of origin and both within and beyond the context of pregnancy.

Both fetal- and placenta-derived messenger RNA transcripts have been detected in maternal plasma, suggesting contributions from both the fetus and placenta to cell-free nucleic acids in maternal circulation [51–53]. While it is possible that fetal tissues and cells release cffDNA into maternal circulation, they are likely not major contributors to total maternal plasma cell-free DNA. Fetal cells such as erythroblasts have previously been hypothesized to be a source of cffDNA. However, fetal cells are present in maternal plasma in significantly lower quantities than cfpDNA (1.2 fetal cells/ml of blood versus 25.4 copies of cfpDNA/ml of plasma) [54,55]. Concentrations of fetal erythroblasts and cfpDNA are not correlated and change independently of each other [48,56]. In vitro release of cffDNA from fetal membranes with fragment sizes consistent with plasma cfpDNA has been demonstrated [32,57]. However, fetal membranes were reported to release less cell-free DNA than placental explants (15.5 ng cell-free DNA/mg of fetal membrane versus 73.7 ng cell-free DNA/mg of placenta after 1 h in culture) [32]. These findings demonstrate that fetal cells are not a major source of cell-free DNA in maternal circulation. It is difficult to differentiate fetal and placental DNA since genetic sequences are typically identical between the two. Distinction relies on comparisons of tissue-specific markers, such as gene expression and DNA methylation, or cases where there is confined placental mosaicism. Whether fetal membranes and other fetal tissues contribute a significant amount of cffDNA in vivo can be further explored by comparing fetal tissue, placental tissue, and maternal plasma cell-free DNA methylation profiles. Notably, the mechanism through which cffDNA and fetal mRNA would enter maternal circulation is unclear. It is unknown how fetal cell-free nucleic acids would cross through placental tissue (i.e., chorionic plate, chorionic villi) and into the intervillous space to access maternal circulation. The amniotic membrane is impermeable to large molecules such as cell-free DNA; therefore, cffDNA present in amniotic fluid (“CffDNA and cfpDNA in other body fluids” section) cannot leave the amniotic sac [58].

Most studies are focused on linear nuclear cfpDNA; however, mitochondrial DNA (mtDNA) is also present in maternal circulation during pregnancy. Quantitative aberrations of circulating mtDNA have been observed in pregnancy complications [59]. Using plasma from surrogate pregnancies, Ma and colleagues [60] demonstrated that placental mtDNA is present in maternal circulation. Placental mtDNA was identified by the presence of single-nucleotide variants that are not carried by the surrogate mother and therefore are specific to the oocyte donor. A majority (88%) of circulating placental mtDNA fragments were linear, in contrast to circulating maternal mtDNA, around 50% of which were circular [60]. Placental extrachromosomal circular DNA (eccDNA) is also present in maternal circulation, identified using placenta-specific single-nucleotide polymorphisms (SNPs) [61]. Fragment size distributions and DNA methylation levels of placental cell-free eccDNA have been characterized and are discussed in later sections of this review.

## Detecting and quantifying cfpDNA

CfpDNA is present in maternal circulation alongside a major fraction of background maternal cell-free DNA mainly derived from white blood cells [4]. Differentiating maternal and placental cell-free DNA remains a challenge. Various methods have been developed to identify and quantify cfpDNA in maternal circulation. Some approaches directly target placenta-specific genetic markers (i.e., Y chromosome sequences [54] and SNPs [62]), while others utilize physical and epigenetic characteristics that differ between placental- and maternal-derived cell-free DNA (i.e., fragment size [63] and DNA methylation [64]). There are 2 metrics that have generally been used for quantifying cfpDNA in the literature: (1) fetal fraction; and (2) concentration. Fetal fraction is the proportion of total plasma cell-free DNA comprised of cfpDNA (% placental DNA) and is routinely measured as a quality control step for NIPT. CfpDNA concentration (copies or genome equivalents per ml of plasma) can be measured using quantitative polymerase chain reaction (qPCR). Methods used for identifying and quantifying cfpDNA are described below and summarized in Table 1.

**Table 1:** Methods for detecting and quantifying cfpDNA.

Method	Test used	Quantification metric
Y chromosome sequences	qPCR [54]	copies/ml
	Whole genome sequencing [3,65–68]	Fetal fraction (%)
SNPs	Whole genome sequencing [62,69–71]	Fetal fraction (%)
	Digital analysis of selected regions (DANSR) [72]	Fetal fraction (%)
Insertion/deletion polymorphisms	Whole genome sequencing [73]	Fetal fraction (%)
DNA methylation	Genome-wide bisulfite sequencing [4]	% placental DNA
	Bisulfite conversion-qPCR [41,74]	copies/ml
	MSRD-MALDI-TOF MS [64]	copies/ml
	MSRD-qPCR [42,75]	copies/ml, genome equivalents/ml
	MSRD-qPCR [76]	Fetal fraction (%)
	MSRD-ddPCR [75]	genome equivalents/ml
	MSRD-ddPCR [77,78]	Fetal fraction (%)
	Single-molecule real-time (SMRT) sequencing [79]	Number of placental DNA fragments
Nucleosome positioning	Whole genome sequencing [80]	Fetal fraction (%)
Sequencing read counts	Whole genome sequencing [81–83]	Fetal fraction (%)
Preferred ends	Paired-end sequencing [84–86]	Fetal fraction (%)
Fragment size	Paired-end sequencing [63]	Fetal fraction (%)
	Semiconductor sequencing [87]	Fetal fraction (%)
	Microchip-based capillary electrophoresis [63,88]	Fetal fraction (%)
Combined methods	Whole genome sequencing [89]	Fetal fraction (%)

### Y chromosome sequences

qPCR quantification of Y chromosome genes including *SRY* and *DYS14* were used in the earliest detection and measurement of cfpDNA [54]. The proportion of reads mapped to the Y chromosome by whole genome sequencing is used for fetal fraction calculation. Various approaches and algorithms have been developed based on this principle [3,65,66,68]. The proportion of Y chromosome reads may be measured relative to the total reads from that plasma cell-free DNA sample and/or compared to the percentage of Y chromosome reads in plasma cell-free DNA from adult males or pregnancies carrying XX fetuses. The major drawback of this method is that it is only applicable for pregnancies carrying XY fetuses.

### SNPs and genetic variation

Placental SNP genotype can be inferred based on knowledge of parental genotypes, allowing direct identification and interrogation of individual cfpDNA fragments. Placenta- and maternal-specific SNP alleles have been used to identify and study cfpDNA separately from background maternal cell-free DNA [45,90]. Placenta-specific SNP alleles are present at SNP loci that are heterozygous in the placental genome and homozygous in the maternal genome, and vice versa for maternal-specific SNP alleles. Fetal fraction can be estimated based on the frequency of sequencing reads carrying placenta-specific SNP alleles [62,69–72,91]. Fetal fraction can also be estimated based on frequencies of insertion/deletion mutations [73].

### DNA methylation

Tissue-specific DNA methylation profiles can be used to classify the tissue of origin of cell-free DNA [92]. Targeting of placenta-specific DNA methylation markers allows for identification of cfpDNA fragments, independent of fetal sex or genotype. Methylation-specific treatment (i.e., methylation-sensitive restriction digestion (MSRD), bisulfite conversion) in combination with DNA quantification methods (i.e., matrix-assisted laser desorption ionization-time of flight mass spectrometry (MALDI-TOF MS) [64], qPCR [41,42,74–76], droplet digital polymerase chain reaction (ddPCR) [75,77,78]) targeting placental differentially methylated regions have been used to quantify cfpDNA. DNA methylation-based tissue of origin deconvolution algorithms have been applied to whole genome bisulfite sequencing data from maternal plasma cell-free DNA samples to estimate cfpDNA proportions [4,93]. Limitations of these methods include sample degradation by bisulfite conversion and dependence on restriction enzyme digestion efficiency. Long-read sequencing platforms (i.e., single-molecule real-time (SMRT) sequencing) allow for bisulfite- and restriction enzyme-free interrogation of DNA methylation status (i.e., the holistic kinetic model) [94]. Yu and colleagues [79] demonstrated the use of long-read sequencing and the holistic kinetic model to identify individual placental and maternal cell-free DNA fragments based on the similarity of their DNA methylation profiles with reference methylomes.

### Fragmentomics

The coverage of the genome by cell-free DNA molecules reflects the nucleosome positioning in their cells of origin (“CfpDNA fragmentomics”—“Genome coverage and nucleosome footprinting” section) [95]. Fetal fraction can be measured based on nucleosome positioning inferred from cell-free DNA sequencing data, according to differential nucleosome positioning between maternal and placental DNA [80]. Various algorithms have been developed for fetal fraction measurement that utilize differential sequencing coverage across genomic regions between cfpDNA and background maternal cell-free DNA [81–83]. Genome coordinates that are overrepresented in cell-free DNA fragment start and end coordinates, termed “preferred ends,” also demonstrate tissue specificity (“CfpDNA fragmentomics”—“Preferred ends” section) and can be used to estimate fetal fraction [84–86].

CfpDNA is on average, shorter than maternal cell-free DNA (“CfpDNA fragmentomics”—“Fragment size” section) [96]. CfpDNA size distributions show a major peak at 143 bp while maternal cell-free DNA size distributions show a major peak at 166 bp [62]. Utilizing this physical difference, fetal fraction can be measured as a function of the ratio of short and long cell-free DNA fragments in a sample [63,87]. Fragment size can be measured using gel electrophoresis [97], qPCR [96], paired-end sequencing [63], capillary electrophoresis [63], or semiconductor sequencing [87]. Size selection can also be used to enrich cfpDNA [97].

### Combined methods

Studies have also demonstrated the benefit of combining predictors to estimate fetal fraction. Gazdarica and colleagues [89] reported improved accuracy of fetal fraction estimates using a model that combines sequence read counts, fragment length, gestational age, maternal body mass index, and DNA library concentration as predictors.

### Absolute cfpDNA quantification

Currently published studies of cfpDNA quantity measure fetal fraction (% placental DNA) or concentration (copies or genome equivalents per ml of plasma). Fetal fraction is affected by both placental and maternal cell-free DNA quantities, and it is difficult to determine which of these factors account for changes in fetal fraction. Therefore, fetal fraction is not a measurement of cfpDNA concentration. Current methods of measuring cfpDNA concentration permit direct investigation of the dynamics of cfpDNA quantity, independent of background maternal cell-free DNA. However, quantities measured using these methods only describe the concentration of the targeted genomic region rather than the total cfpDNA concentration in maternal plasma. The latter would involve inferring total cfpDNA concentration based on the quantity of a targeted region, which works under the assumption that all genomic regions are equally represented in circulating cell-free DNA. Individual fragment-level identification and quantification of cfpDNA beyond placenta-specific genetic and methylated loci requires further characterization of tissue-specific characteristics of cell-free DNA. Absolute quantification of cfpDNA (nanograms or picomoles of DNA) would allow for direct characterization of cfpDNA quantities across gestation and in pregnancy pathologies. Absolute quantification of cell-free DNA can be achieved by using spike-in controls during sequencing protocols [98,99].

## CfpDNA release and clearance

### Apoptosis

Apoptosis is involved in the normal development and cellular turnover of the placenta [100] and it is hypothesized that this contributes cfpDNA into maternal circulation. Mechanistic links between apoptosis and cfpDNA release have been investigated in vitro using placental explant studies. Parallel increases in apoptosis and cfpDNA release over time in mouse placental explants under physiologic oxygen levels (8% O_2_) have been reported [29]. Hypoxia-induced oxidative stress (hypoxia (0.5% O_2_) followed by reoxygenation (10% O_2_)) was found to significantly increase apoptosis and cfpDNA release, compared to normoxic conditions (10% O_2_) in human term placental explants [28]. Hydrogen peroxide- and hyperoxia (21% O_2_)-induced oxidative stress did not increase apoptosis or cfpDNA release in mouse placental explants [29]. One study reported significantly increased apoptosis and cfpDNA release in mouse placental explants following stimulation of inflammation by lipopolysaccharide [29], while other studies found no increase in either apoptosis nor cfpDNA release in human term placental explants following lipopolysaccharide treatment [30,31]. Induction of sterile inflammation in human placental explants significantly increased cfpDNA release without a significant increase in apoptosis, indicating non-apoptotic contributions to cfpDNA release [31]. Apoptosis stimulation (doxorubicin) significantly increased apoptosis and cfpDNA release in human term placental explants [31]. Suppression of apoptosis by caspase inhibition significantly decreased apoptosis and cfpDNA release in mouse placental explants [29]. These studies demonstrate that apoptosis, among other release mechanisms, generates cfpDNA and that factors inducing apoptosis can lead to increased cfpDNA shedding from the placenta. Notably, placental hypoxia and inflammation are characteristics of preeclampsia [101,102]. Increased placental apoptosis in response to these factors may contribute to the increased cfpDNA levels in preeclampsia [47]. The presence of cfpDNA in apoptosis-derived placental extracellular vesicles (syncytiotrophoblast microparticles and apoptotic bodies) that were generated in vitro [103,104] or collected from maternal plasma [103,105] provide further evidence for the apoptotic release of cfpDNA. The fragment size of cfpDNA (∼150 to 200 bp) is consistent with the conserved DNA fragmentation patterns of apoptosis. Specifically, the modal fragment size of cfpDNA (143 to 200 bp) is equivalent to the length of DNA that wraps around a nucleosome, reflecting the internucleosomal cleavage that occurs during apoptosis [96,106].

### Necrosis

Necrosis is known to be another release mechanism of cell-free DNA. Placental necrosis is implicated in pregnancy pathologies such as preeclampsia [107]. Whether necrosis contributes to the increased cfpDNA in preeclamptic pregnancies [47] and the extent of this contribution is unknown. In vitro studies investigating cfpDNA release from placental explants have suggested necrosis-mediated cfpDNA generation [28,29,103]. In contrast to apoptosis, necrosis involves random DNA fragmentation and is believed to generate longer cell-free DNA fragments (>10,000 bp) [108]. The relationship between fragment length and cell death mechanism (apoptosis versus necrosis) may not be as clear cut as it is presented in the literature. Long-read sequencing has uncovered a significant quantity of long plasma cell-free DNA (>500 bp) that was previously undetectable using short-read sequencing platforms [79,109]. Notably, long cell-free DNA shows size distribution patterns (200 bp periodicity) reflective of multi-nucleosomal units, therefore consistent with apoptotic internucleosomal DNA fragmentation [79,109]. Therefore, the relationships between cell-free DNA fragmentation patterns and cell death mechanisms need to be further investigated.

### Exosomes

Exosomes are actively released by cells to carry out intracellular communication and are another source of cell-free DNA [110]. Placental exosomes can be detected in maternal circulation from the sixth week of gestation and quantities increase with gestational age [111,112]. Placental exosomes are identified by detection of the surface protein, placental alkaline phosphatase (PLAP) [112,113]. Y chromosome sequences can be detected in exosome-bound DNA isolated from maternal plasma, demonstrating the presence of exosome-bound cfpDNA in maternal circulation, although concentrations of exosome-bound cfpDNA were on average, 10 to 11 times lower than free cfpDNA concentrations [114–116]. Exosomes are attractive sources of cfpDNA for NIPT due to the increased stability of extracellular vesicle-bound cell-free nucleic acids [117]. Preliminary studies have demonstrated successful noninvasive fetal sex determination, RHD genotyping, and detection of fetal trisomy and monogenic diseases using exosomal DNA isolated from maternal plasma [114,118]. Placental exosomes have been suggested to be involved in the onset of parturition [119–121]. Whether the cfpDNA encapsulated in placental exosomes is involved in this process is unknown.

### CfpDNA clearance

Following delivery, cfpDNA is rapidly cleared from maternal plasma [122,123]. CfpDNA clearance occurs in a biphasic pattern. The first phase takes place during the first 2 h after delivery and involves rapid clearance, with an average cfpDNA half-life of 1 h [123]. The second phase involves slower clearance, with an average cfpDNA half-life of 13 h [123]. This pattern suggests the involvement of different cfpDNA clearance mechanisms across the 2 phases [123]. CfpDNA generally reaches undetectable levels by 2 days postpartum [123]. Cell-free DNA clearance is believed to be mediated by plasma endonucleases and organs such as the liver, kidney, and spleen [124]. Plasma endonucleases and transrenal clearance were found to both be minor contributors to the elimination of cfpDNA [123]. CfpDNA clearance is compromised in preeclampsia, as demonstrated by increased cfpDNA half-lives (median half-life of 114 min in preeclampsia versus 28 min in healthy controls) and persistence of cfpDNA postpartum at time points when normally undetectable in maternal plasma [125]. This clarifies a mechanism for the increased cfpDNA levels observed in preeclampsia [47] and provides support for roles of the kidneys and liver, which are affected by preeclampsia, in normal cfpDNA clearance. Further research on the clearance mechanisms of cfpDNA is required. Placental cell-free eccDNA also demonstrates rapid postpartum clearance from maternal plasma and has comparable half-lives to linear cfpDNA [126].

### CfpDNA release and clearance mechanisms: Future research directions

Despite widespread clinical implementation, the exact mechanisms underlying cfpDNA generation and release remain largely unknown. Further in vitro and in vivo studies directly studying cfpDNA release are required to more definitively identify the cell death and release mechanisms involved, the proportions which they contribute, and how these change across different conditions. Other forms of cell death aside from apoptosis and necrosis, such as pyroptosis [127,128], ferroptosis [129], aponecrosis [103], and necroptosis [130] have been implicated in placental dysfunction and should also be investigated as potential sources of cfpDNA. In vivo studies will be beneficial for understanding dynamics of cfpDNA release and clearance and how the balance between these processes affect cfpDNA levels across gestation and in pregnancy pathologies. Knowledge of “normal” cfpDNA characteristics and dynamics in healthy pregnancies is required to properly characterize aberrant patterns in pregnancy complications. Characterization of the specific cfpDNA features generated by different release mechanisms can be leveraged to provide real-time insights into the state of the placenta. This can be a powerful research tool for longitudinal studies of placental development and disease, which are currently hindered by ethical constraints and limited availability of early- and mid-gestation tissue samples. This will open avenues for many more clinical applications of cfpDNA beyond the current NIPT repertoire, which is limited to genetic screening of the fetus. Overall, an improved understanding of the basic biological mechanisms underlying cfpDNA is required to fully leverage it as a tool for assessing placental health and screening for complex pregnancy complications.

## CfpDNA genomics

CfpDNA is representative of the full placental genome [62], providing a means for noninvasive genome-wide characterization of the placenta. NIPT has been widely implemented to screen for common fetal aneuploidies including Trisomies 13, 18, and 21 [131] by assessing for overrepresentation of the affected chromosomes in maternal plasma cell-free DNA [3,132]. The effectiveness of NIPT for aneuploidy screening has recently been reviewed elsewhere [133–135].

Efforts are being made to expand NIPT to include the detection of sub-chromosomal aberrations such as copy number variations and microdeletions [87,135–137]. Methods for detecting fetal de novo mutations, paternal inheritance, and maternal inheritance for the diagnosis of monogenic disorders have been summarized elsewhere [138,139], and these methods have been used in efforts to reconstruct full placental genomes [62,84,140,141]. The ongoing development of long-read sequencing platforms will open opportunities for detecting larger-scale genomic aberrations such as recombination events, structural variants, and repeat expansions [79,109,142].

Most studies using cfpDNA to noninvasively investigate the placental genome are focused on NIPT applications. Notably, they are geared towards using cfpDNA to determine fetal genotypes. While fetal and placental genome sequences are generally identical, confined placental mosaicism can occur [33]. In such cases, the fetal and placental genomes do not match, highlighting the importance of making the distinction between cell-free “fetal” DNA and cell-free “placental” DNA, especially when discussing clinical applications of cfpDNA. A future research direction is to explore the use of cfpDNA for noninvasive investigation of placental genomic aberrations in the context of placental dysfunction and pregnancy complications [143].

## CfpDNA methylation

DNA methylation is an epigenetic modification involved in the modulation of gene expression. It demonstrates tissue specificity [144] and plays an important role in development, health, and disease [145,146].

The placenta methylome is characterized by global hypomethylation, tissue-specific methylated CpG islands, large spans of intermediate methylation called partially methylated domains, and placenta-specific imprinted regions [147]. Studies have demonstrated concordance between cfpDNA and placental methylomes [45,46,148]. Lun and colleagues [45] used whole genome bisulfite sequencing to construct a cfpDNA methylome from a maternal plasma cell-free DNA sample by sub-setting cell-free DNA reads carrying placenta-specific alleles at informative SNP loci. Similarly, they constructed the “shared” methylome by identifying cell-free DNA reads containing alleles carried by both the fetus and mother at informative SNP loci [45]. Consistent with known patterns of placental DNA methylation, overall DNA methylation levels were lower in cfpDNA relative to shared cell-free DNA, and increased with gestational age [45]. This pattern was observed genome-wide, across repeat and non-repeat regions, autosomes, and the X chromosome [45]. CpG methylation densities were correlated between cfpDNA and placenta, and the shared methylome and maternal blood cells [45]. This is consistent with the placental origin of cfpDNA and the hematopoietic origin of background maternal cell-free DNA [26,46]. At the time of writing this review, this is the only publication that has separated cfpDNA from background maternal cell-free DNA to study cfpDNA methylation. Consistent with these findings, Jensen and colleagues [46] reported reduced genome-wide CpG methylation in total maternal cell-free DNA compared to non-pregnant cell-free DNA using whole genome bisulfite sequencing. Pregnant and non-pregnant cell-free DNA were differentially methylated in regions that were also differentially methylated between placenta and non-pregnant cell-free DNA [46]. Chu and colleagues [148] reported similar findings using targeted bisulfite sequencing in first trimester pregnant and non-pregnant cell-free DNA samples. Their study revealed a reduction in overall cell-free DNA CpG methylation in pregnant plasma compared to non-pregnant plasma. The identity, magnitude, and directionality of differentially methylated CpGs identified between pregnant cell-free DNA and non-pregnant cell-free DNA were consistent with those identified when comparing chorionic villus and maternal leukocyte DNA [148]. More dramatic differences were observed in intron and exon regions compared to regulatory elements, consistent with previous reports of increased DNA methylation in regulatory elements in placental DNA, despite global hypomethylation compared to other tissues [148]. Placenta-derived cell-free eccDNA displays lower methylation density compared to maternal-derived cell-free eccDNA, consistent with the patterns observed in linear cfpDNA [126].

DNA methylation-based tissue of origin deconvolution algorithms have been developed using reference methylomes for different tissues to estimate proportions of tissue contributions to circulating cell-free DNA. Inclusion of placenta reference methylomes allows for estimation of the proportion of cfpDNA in maternal plasma and investigation of how relative tissue contributions to cell-free DNA change across gestation and in pregnancy pathologies [4,93,149]. DNA methylation patterns can also be used to identify the placental or maternal origin of individual cell-free DNA molecules sequenced using long-read sequencing platforms [79].

Identification of DNA methylation markers for the detection of cfpDNA has been an active area of research ever since its discovery. Such markers are applicable for identifying cfpDNA in all pregnancies regardless of fetal sex and genotype. Evidence points towards the hematopoietic origin of background maternal cell-free DNA [4,150], therefore, genomic regions that are differentially methylated between placenta and white blood cells are investigated as candidate markers. Table 2 lists cfpDNA methylation markers that have been validated in maternal plasma. CfpDNA carries placental genome imprinting status, and this has also been proposed as a method for differentiating cfpDNA from background maternal cell-free DNA [45,151].

**Table 2:** CfpDNA methylation markers validated in maternal plasma.

Marker	Genomic coordinates	Citation
**Hypomethylated**		
*PDE9A* (“CGI084”)	chr21:43,000,285–43,000,711 (genome assembly not specified)^a^	Validated by Chim and colleagues [152]; genome coordinates extracted from Yamada and colleagues [153]
“CGI137”	chr21:46,281,910–46,282,569 (genome assembly not specified)^a^	Validated by Chim and colleagues [152]; genome coordinates extracted from Yamada and colleagues [153]
**Hypermethylated**		
*RASSF1A* promoter	chr3:50340551–50342500 (GRCh37/hg19)^c^	Chan and colleagues [42]
*STAT5A* promoter	chr17:42287439–42289714 (GRCh37/hg19)^c^	Rahat and colleagues [154]
*DSCR3*	chr21:38629500–38629559 (genome assembly not specified)^a^	Kim and colleagues [155]
*ERG* promoter	chr21:38506973–38507096 (GRCh38/hg38)^b^	Chen and colleagues [156]
*VAPA-APCDD1* intergenic region	chr18:10022563–10023955 (NCBI36/hg18)^a^	Tsui and colleagues [157]
*HLCS* promoter	chr21:37,274,651–37,275,063 (NCBI36/hg18)^a^	Tong and colleagues [43]
“DMR 5”	chr1:201709481–201709561 (GRCh37/hg19)^b^	Ioannides and colleagues [77]
*ERG* intragenic region (“FSMR-E”)	ch21:39865824–39865906 (GRCh37/hg19)^a^	Lim and colleagues [158]
*UMODL1* Promoter (“FSMR-U1”)	ch21:43482788–43482892 (GRCh37/hg19)^a^	Lim and colleagues [158]
*UMODL1* Promoter (“FSMR-U2”)	ch21:43483864–43483956 (GRCh37/hg19)^a^	Lim and colleagues [158]
“EP6”	chr21:42062534–42062637 (GRCh38/hg38)^b^	Lim and colleagues [159]
“EP7”	chr21:42064037–42064163 (GRCh38/hg38)^b^	Lim and colleagues [159]
*CD99* Intron 1 (“EF3”)	chrX:2624944–2626149 (genome assembly not specified)^a^	Della Ragione and colleagues [160]

^a^Genome coordinates stated in cited publication. Whether genome coordinates are 0- or 1-based was not specified.

^b^Identified genome coordinates by searching up forward and reverse PCR primers using UCSC Genome Browser Human BLAT Search. Genome coordinates are 1-based.

^c^Genomic regions annotated to GENCODE v33. Genome coordinates are 1-based.

CfpDNA methylation carries great potential as a tool for research and noninvasive screening. Utilizing cfpDNA to characterize and detect disease-specific placental DNA methylation changes across gestation is a future research direction that would advance the understanding of placental pathologies and greatly broaden the potential applications of NIPT [161–163]. This is significant since it is difficult to obtain placental tissue samples during pregnancy for research and screening purposes as this requires invasive procedures such as chorionic villus sampling. Investigation of other epigenetic modifications, such as hydroxymethylation, in cfpDNA is another research direction to pursue [164].

## Circulating placental nucleosomes and histone modifications

Cell-free DNA can circulate in multiple forms: naked DNA, circulating nucleosomes, or bound to extracellular vesicles, proteins, and histones [114,165]. Circulating nucleosomes and associated histone modifications have been studied as noninvasive biomarkers for cancer, liver transplant rejection, and idiopathic pulmonary fibrosis [166–168]. Histone modifications play roles in placental development and pregnancy complications [169–173]. Investigation of circulating placental nucleosomes and associated histone modifications can provide tools for studying placental health and development noninvasively throughout gestation. Bouvier and colleagues [174] demonstrated that circulating nucleosome levels in maternal plasma increases with gestational age and in preeclampsia, although proportions of maternal- and placenta-derived nucleosomes were unknown. While knowledge of circulating placental nucleosomes in maternal plasma is limited, information on placental nucleosome positioning is carried by cfpDNA (“CfpDNA fragmentomics”—“Genome coverage and nucleosome footprinting” section). Patterns of cell-free DNA fragmentation are reminiscent of nucleosome structure [62,79,85], and genome coverage by cell-free DNA is informative of nucleosome positioning in the tissue of origin [95].

## CfpDNA fragmentomics

Fragmentomics is a new area of research that studies the mechanisms underlying cell-free DNA fragmentation and the specific characteristics that are generated. As demonstrated by tissue- and disease-specific fragmentomic patterns, the process of cell-free DNA fragmentation is non-random. Fragmentomic characteristics that have been identified and studied include fragment size, end motifs, genome coverage, preferred ends, and jagged ends.

### Fragment size

The fragment size distribution of cfpDNA is distinct from that of background maternal cell-free DNA. CfpDNA is generally shorter than background maternal cell-free DNA, made evident by a shift towards a shorter size distribution and depletion of longer fragments [3,62,63,96,97,175]. This trend is observed not only in linear nuclear cfpDNA, but also circulating eccDNA [61] and mtDNA [60]. The size distribution of background maternal cell-free DNA shows a major peak at 166 bp, corresponding to the length of DNA wrapped around a nucleosome core (∼147 bp) plus adjacent linker regions (modal length of ∼20 bp) [62,176]. The cfpDNA size distribution shows a reduced 166 bp peak and a major peak is observed at 143 bp instead [62]. This corresponds to the length of DNA wrapping around a nucleosome core only [62,176]. The 10 bp periodicity observed below 150 bp in cfpDNA is hypothesized to be due to trimming of nucleosome-bound DNA by nucleases [62,176]. A minor peak at approximately 320 bp has also been observed in cfpDNA size distributions, likely representing dinucleosomal units [79,175].

Using long-read sequencing, Yu and colleagues [79] discovered a population of long cell-free DNA (>500 bp) in maternal plasma. Detection of these fragments is typically beyond the technical limits of short-read sequencing platforms. Long cell-free DNA fragments were present in both maternal and placental cell-free DNA pools, which were distinguished using SNP genotyping [79]. Consistent with previous observations of cfpDNA being shorter than background maternal cell-free DNA, the proportion of long cell-free DNA was reduced in cfpDNA compared to background maternal cell-free DNA [79]. Long fragments comprised greater than 20% of cfpDNA and this proportion increased with gestational age [79]. The longest cfpDNA molecule was 23,635 bp [79]. Long cell-free DNA (>250 bp) can be recovered using DNA repair prior to sequencing [142]. Notably, this process enriches cfpDNA but not background maternal cell-free DNA, pointing towards differences in the types of DNA damage between placental and maternal cell-free DNA, and differences in the mechanisms underlying their fragmentation [142]. While often stated in the literature that apoptosis generates short cell-free DNA while necrosis generates long cell-free DNA [108], findings from the recent long-read sequencing studies may indicate otherwise. Size distribution peaks corresponding to multi-nucleosomal units suggest apoptotic origins of long cell-free DNA [109]. Therefore, the relationships between cell-free DNA fragment size and cell death mechanisms remain poorly understood.

Cell-free DNA fragment size and DNA methylation levels are positively correlated [45,46,126,177,178]. Total maternal plasma cell-free DNA methylation and fragment size both increase with gestational age [177], consistent with previous observations of placental DNA methylation increasing with advancing gestational age [179]. These findings also point towards the role of DNA methylation in regulating cell-free DNA fragmentation and generating the distinct size distribution of cfpDNA. The role of DNA methylation in cell-free DNA fragmentation may be related to nucleosome packaging and nuclease activity (i.e., DNASE1L3) [178].

Clinical applications of cfpDNA fragment size are being investigated. The size distribution of cell-free DNA is altered in preeclampsia, with a decrease in the proportion of long fragments [79,180,181]. Whether this decrease is in background maternal cell-free DNA, cfpDNA, or both has not been determined. Researchers were able to identify pregnancies with preeclampsia based on the proportion of long cell-free DNA (>170 bp) in maternal plasma using long-read sequencing [79] and ddPCR [180]. The distinct size distributions of background maternal cell-free DNA and cfpDNA have been investigated for NIPT applications such as fetal fraction measurement [63] and cfpDNA enrichment [97,182].

### Fragment end motifs

Cell-free DNA fragment end motifs are informative of the nucleases and fragmentation processes by which they were generated. Associations between specific fragment end motifs and nucleases have been identified. DNASE1L3, DNASE1, and DFFB have been found to have preferences to cleave 5’ of C, T, and A nucleotides, respectively [183–185]. End motifs are also associated with cell-free DNA fragment size, likely due to the stepwise activity of endonucleases [79,184]. Shorter cell-free DNA fragments (<1,000 bp) predominantly carry C- and G- ends while longer fragments (>1,000 bp) predominantly carry A- and G-ends [79]. The frequency of T- and C-ends decrease as fragment size increases beyond 1,000 bp [79].

Studies have demonstrated that cell-free DNA end motif profiles are associated with tissue of origin and are altered in pathological states such as cancer, likely attributable to changes in nuclease activities and DNA fragmentation processes [90,183]. CfpDNA end motif profiles are distinct from those of background maternal cell-free DNA [90]. The relative contributions of an end motif profile that is hypothesized to be generated by non-enzymatic DNA fragmentation was elevated in cfpDNA in the first trimester compared to the second and third trimesters [183]. This is consistent with the state of increased oxidative stress in the early placenta [183]. Yu and colleagues [79] demonstrated that the cell-free DNA end motif profile of patients with preeclampsia is distinct from control subjects. A reduction in G- and A-ends and increase in T- and C-ends was reported [79]. These observations are consistent with what would be expected given the relationship between cell-free DNA fragment size and end motifs, and the observation of a decreased proportion of long cell-free DNA in preeclampsia [79]. Upon investigating expression levels of endonucleases, DNASE2 was found to be up-regulated in preeclamptic placentas, suggesting a potential role in aberrant cell-free DNA fragmentation in preeclampsia [79]. The relationship between DNASE2 activity and cell-free DNA fragment end motifs has not yet been investigated due to the lethality of *Dnase2a* deletion in mouse models [79]. The authors developed a classifier to identify preeclamptic pregnancies based on cell-free DNA 4-mer end motifs [79]. This demonstrates the potential application of fragment end motifs as an indicator of placental health and a noninvasive biomarker for pregnancy complications.

### Genome coverage and nucleosome footprinting

Cell-free DNA fragmentation is influenced by nucleosome structure and positioning. This is made evident through cell-free DNA size distribution patterns (“CfpDNA fragmentomics”—“Fragment size” section) and the observed relationships between cell-free DNA genome coverage, nucleosome positioning, and gene expression.

Cell-free DNA genome coverage reflects the nucleosome structure and positioning in tissues of origin. This is based on the principle that nucleosome-bound DNA is protected from nuclease activity and is expected to be more abundantly represented in cell-free DNA. On the other hand, naked DNA is more susceptible to nuclease digestion and is expected to have lower representation in cell-free DNA [95]. Accordingly, genome mapping of cell-free DNA sequencing reads shows peaks of increased coverage that demonstrate a ∼190 bp periodicity, consistent with the length of DNA wrapping around a nucleosome plus the linker regions [85,95,176,186]. Based on this principle, patterns of cell-free DNA coverage are consistent with the expected nucleosome positioning in specific genomic regions (i.e., transcriptional start sites [95,187], nucleosome depleted regions [176], open and closed chromatin regions [85,95], and transcription factor binding sites [95]). Cell-free DNA coverage therefore reflects tissue of origin [85,95] and gene expression activity [95,176]. Cell-free DNA coverage also footprints transcription factor binding [95].

Tissue-specific nucleosome positioning inferred using cell-free DNA coverage can measure the fetal fraction in maternal plasma cell-free DNA samples. Straver and colleagues [80] developed a method for estimating fetal fraction based on the proportions of cell-free DNA fragments starting at different positions in the nucleosome structure, according to cfpDNA-specific patterns. Sun and colleagues [85] developed a metric called the “orientation-aware cell-free DNA fragmentation (OCF)” value, which measures tissue contributions to total plasma cell-free DNA based on representation levels of tissue-specific open chromatin region nucleosome positioning. In maternal plasma cell-free DNA, the T cell OCF value was decreased while the placenta OCF value was increased compared to non-pregnant plasma cell-free DNA samples [85].

Gene expression is influenced by nucleosome positioning. Therefore, cell-free DNA coverage can be used to deduce nucleosome positioning at promoter regions, which can in turn infer gene expression patterns in the tissue of origin. Accordingly, cell-free DNA coverage at promoter regions has been found to be inversely associated with gene expression levels [188]. Han and colleagues [187] demonstrated the feasibility and utility of noninvasively inferring placental gene expression using maternal plasma cell-free DNA. The authors reported reduced coverage of placenta-specific genes in pregnant plasma cell-free DNA compared to non-pregnant plasma cell-free DNA [187]. Gestational age-dependent changes in transcription start site coverage of placenta-specific genes (corticotropin releasing hormone (*CRH*) and chorionic gonadotropin beta polypeptide 5 (*CGB5*)) in maternal plasma cell-free DNA consistent with their respective gene expression dynamics across gestation were observed [187]. Dynamics of gene isoform expression across gestation could be characterized [187]. Classifiers were built for determining gestational trimester, fetal sex, and trisomy 21 status based on inferred gene expression from maternal plasma cell-free DNA coverage of transcription start sites and transcription factor binding sites [187]. Other researchers have developed classifiers for identifying pregnancies with preeclampsia, gestational diabetes mellitus, fetal growth restriction, and macrosomia based on nucleosome positioning and gene expression deduced using maternal plasma cell-free DNA genome coverage [189–191]. Overall, mapping of nucleosome positioning using cell-free DNA provides a noninvasive method for studying maternal and placental gene expression profiles across gestation [187,192].

### Preferred ends

The genomic coordinates at which cell-free DNA fragments start and end are non-random. Fragment end coordinates that are overrepresented in cell-free DNA have been termed “preferred ends.” Preferred ends are associated with tissue of origin [84] and fragment size [86].

Chan and colleagues [84] identified distinct preferred ends for maternal and placental cell-free DNA. They demonstrated that the ratio of fragments carrying placental- and maternal-specific preferred ends was positively correlated with fetal fraction measured using the proportion of Y chromosome reads [84]. The size distribution of fragments carrying the placenta-specific preferred ends was shorter than that of fragments carrying the maternal-specific preferred ends, reflecting the different size distributions of cfpDNA and background maternal cell-free DNA [84]. Notably, tissue-specific preferred ends identified in one pregnancy were robust across samples from other pregnancies [84].

Sun and colleagues [86] identified size-specific preferred ends after separating sequence reads into short (60 to 155 bp) and long (170 to 250 bp) cell-free DNA fragment categories. The ratio of fragments carrying short and long fragment-specific preferred ends was positively correlated with fetal fraction measured using the proportion of Y chromosome reads [86]. Enriching cfpDNA using short fragment-specific preferred ends improved the performance of trisomy 21 detection [86]. Positioning of short and long fragment-specific preferred ends were associated with nucleosome structure, with short fragment-specific preferred ends often being located within the nucleosome core and long fragment-specific preferred ends often being located in the linker regions [86]. This provides further evidence for the role of nucleosome positioning in mediating cell-free DNA fragmentation patterns.

### Jagged ends

The term “jagged ends” describes single-stranded overhangs that are present on most cell-free DNA fragments (87.7% of plasma cell-free DNA fragments from a cohort of 15 pregnant women) [193]. Patterns of jagged end lengths are reminiscent of nucleosome positioning [193]. The jagged end length of cell-free DNA has been hypothesized to be associated with tissue of origin. Accordingly, cfpDNA bears longer jagged ends than background maternal cell-free DNA and jagged end length was positively correlated with fetal fraction [193]. DNASE1, an endonuclease involved in cell-free DNA fragmentation, is implicated in the generation of jagged ends [193]. Consistent with the increased jaggedness of cfpDNA, DNASE1 is more highly expressed in the placenta than in white blood cells [193].

## CffDNA and cfpDNA in other body fluids

CffDNA and cfpDNA have been detected in maternal body fluids other than blood such as cerebrospinal fluid [194] and peritoneal fluid [58,195].

Several studies have reported the presence of cell-free DNA carrying Y chromosome sequences or placenta-specific SNP alleles in maternal urine [196–199]. It can be assumed that this is cfpDNA filtered from maternal blood. Fragment size analysis has revealed that urinary cfpDNA is highly fragmented, with size distribution peaks between 29 to 45 bp [199]. CfpDNA is present in lower amounts in maternal urine compared to maternal plasma. The fetal fraction in third trimester maternal urine samples was 1.92% to 4.73%, while the fetal fraction in maternal plasma at the same gestational age was around 20.4% [199].

There is a large fraction of cffDNA present in amniotic fluid. The concentration of cffDNA is 100- to 200-fold higher in amniotic fluid compared to the concentration of cfpDNA in maternal plasma [200] and is correlated with gestational age [201]. Burnham and colleagues [202] found that amniotic fluid cffDNA is highly fragmented and shorter than plasma cfpDNA, with a median fragment size of 108 bp. Analysis of fragmentation patterns in euploid and aneuploid pregnancies revealed fragmentation profiles unique to different karyotypes [201,203]. Amniotic fluid cffDNA is mainly derived from the fetus rather than the placenta, indicated by the lack of correlation with plasma cfpDNA levels [200], absence of placenta-specific mRNA transcripts [204], and low detection of hypermethylated *RASSF1A*, a placenta-specific DNA methylation pattern [205], in amniotic fluid.

## CfpDNA function

Whether cfpDNA plays a functional role during pregnancy is unknown. Sterile inflammation is implicated in parturition and pregnancy complications such as preeclampsia [206,207]. Given that cfpDNA is hypomethylated like microbial DNA, it is hypothesized that cfpDNA can act as a proinflammatory danger-associated molecular pattern and activate Toll-like Receptor 9 (TLR9) [30,208]. Increases in cfpDNA levels near delivery [55], in preterm birth [209], and in inflammation-associated placental pathologies (i.e., preeclampsia) [47] provide further rationale for exploring this hypothesis. In vitro studies have investigated the proinflammatory potential of cell-free DNA released from mouse and human placental explants. Cell-free DNA isolated from explant culture media has been found to induce cytokine secretion from mouse macrophages [29] and human maternal peripheral blood mononuclear cells (PBMCs) [208]. Kazemi and colleagues [208] found that cell-free DNA from human maternal plasma collected at 36 to 38 weeks of gestation induced cytokine secretion from maternal PBMCs, while cell-free DNA from maternal plasma collected postpartum did not. This suggests a proinflammatory role specific to cfpDNA. Cell-free DNA specifically triggered monocytes, which then amplified the immune response by activating neighboring T-cells [208]. They also reported that cell-free DNA only triggered immune cells when transfected in lipid-based transfection reagents, suggesting that extracellular vesicle-bound cfpDNA may specifically be responsible for cfpDNA-mediated inflammation. This is likely related to increased efficiency of cellular uptake and signaling [208]. Active release, protection, and transfection of cfpDNA through extracellular vesicles (i.e., exosomes), rather than passive release through cell death, would be more consistent with a functional role of cfpDNA. The culture media of PBMCs treated with cell-free DNA promoted endothelial cell activation and myometrial contraction, which are both processes involved in parturition [208]. In contrast with the above reports, one study found that cell-free DNA collected from human term placenta culture media did not induce cytokine release from maternal PBMCs [30]. Further investigation of DNA methylation patterns revealed that the collected cell-free DNA was not hypomethylated, making it an unsuitable TLR9 agonist [30]. Further in vitro and in vivo studies will provide more insights into any potential biological roles of cfpDNA.

## Future directions

Great progress has been made in developing the clinical utility of cell-free DNA, from NIPT to cancer monitoring. However, many gaps remain in the understanding of the release mechanisms, form, and functions of cell-free DNA. This hinders the potential of cell-free DNA not only as a clinical tool, but also as a research tool for better understanding tissue health, development, and disease. Further investigation of the basic biology of cell-free DNA and characterization of its molecular characteristics will allow researchers to collect more information about the states of their tissues of origin. The ongoing development of in vitro models, sequencing technologies, and bioinformatic tools will help to drive these advancements and open doors for future research directions and subsequent development of clinical applications. Insights into the form and physical properties of cell-free DNA can guide improvements in isolation and analysis methods. General characterization of the effects of physiological and lifestyle factors [210] on cell-free DNA will help deconvolute cell-free DNA signals and increase the power of pathology-specific analyses. Reproductive and women’s health research is underfunded [211,212] and studies investigating cell-free DNA in pregnancy are limited compared to other fields such as cancer. Directing more attention to studying cell-free DNA in pregnancy will improve our understanding of the placental physiological processes that generate cfpDNA, the functional role of cfpDNA in pregnancy, and how cfpDNA can be leveraged to study and monitor placental health and disease. It is also of value to study background maternal cell-free DNA to gain insights into maternal adaptations to pregnancy and any maternal origins of pregnancy complications and the impacts of these conditions on maternal health. Ultimately, this will lead to improvements in perinatal care and reproductive and women’s health.

## References

[1] Lo YD, Corbetta N, Chamberlain PF, Rai V, Sargent IL, Redman CW, et al. Presence of fetal DNA in maternal plasma and serum. Lancet. 1997;350(9076):485–487. doi: 10.1016/S0140-6736(97)02174-0 9274585

[2] Karakas B, Qubbaj W, Al-Hassan S, Coskun S. Noninvasive digital detection of fetal DNA in plasma of 4-week-pregnant women following in vitro fertilization and embryo transfer. PLoS ONE. 2015;10(5):e0126501. doi: 10.1371/journal.pone.0126501 25970589 PMC4430227

[3] Fan HC, Blumenfeld YJ, Chitkara U, Hudgins L, Quake SR. Noninvasive diagnosis of fetal aneuploidy by shotgun sequencing DNA from maternal blood. Proc Natl Acad Sci U S A. 2008;105(42):16266–16271. doi: 10.1073/pnas.0808319105 18838674 PMC2562413

[4] Sun K, Jiang P, Chan KA, Wong J, Cheng YK, Liang RH, et al. Plasma DNA tissue mapping by genome-wide methylation sequencing for noninvasive prenatal, cancer, and transplantation assessments. Proc Natl Acad Sci U S A. 2015;112(40):E5503–E5512. doi: 10.1073/pnas.1508736112 26392541 PMC4603482

[5] Ji L, Brkić J, Liu M, Fu G, Peng C, Wang YL. Placental trophoblast cell differentiation: physiological regulation and pathological relevance to preeclampsia. Mol Aspects Med. 2013;34(5):981–1023. doi: 10.1016/j.mam.2012.12.008 23276825

[6] Wang Y, Zhao S. Vascular Biology of the Placenta. 2010. 21452443

[7] Burton G, Charnock-Jones D, Jauniaux E. Regulation of vascular growth and function in the human placenta. Reproduction. 2009;138(6):895–902. doi: 10.1530/REP-09-0092 19470597

[8] Moser G, Windsperger K, Pollheimer J, de Sousa Lopes SC, Huppertz B. Human trophoblast invasion: new and unexpected routes and functions. Histochem Cell Biol. 2018;150:361–370. doi: 10.1007/s00418-018-1699-0 30046889 PMC6153604

[9] Roberts VH, Morgan T, Bednarek P, Morita M, Burton G, Lo J, et al. Early first trimester uteroplacental flow and the progressive disintegration of spiral artery plugs: new insights from contrast-enhanced ultrasound and tissue histopathology. Hum Reprod. 2017;32(12):2382–2393. doi: 10.1093/humrep/dex301 29136193 PMC6251668

[10] Brett KE, Ferraro ZM, Yockell-Lelievre J, Gruslin A, Adamo KB. Maternal–fetal nutrient transport in pregnancy pathologies: the role of the placenta. Int J Mol Sci. 2014;15(9):16153–16185. doi: 10.3390/ijms150916153 25222554 PMC4200776

[11] Burton GJ, Fowden AL. The placenta: a multifaceted, transient organ. Philos Trans R Soc Lond B Biol Sci. 2015;370(1663):20140066. doi: 10.1098/rstb.2014.0066 25602070 PMC4305167

[12] Steegers EA, Von Dadelszen P, Duvekot JJ, Pijnenborg R. Pre-eclampsia. Lancet. 2010;376(9741):631–644. doi: 10.1016/S0140-6736(10)60279-6 20598363

[13] Roberts JM, Rich-Edwards JW, McElrath TF, Garmire L, Myatt L, Collaboration GP. Subtypes of preeclampsia: recognition and determining clinical usefulness. Hypertension. 2021;77(5):1430–1441. doi: 10.1161/HYPERTENSIONAHA.120.14781 33775113 PMC8103569

[14] Benton SJ, Leavey K, Grynspan D, Cox BJ, Bainbridge SA. The clinical heterogeneity of preeclampsia is related to both placental gene expression and placental histopathology. Am J Obstet Gynecol. 2018;219(6):604–e1. doi: 10.1016/j.ajog.2018.09.036 30278173

[15] Jung E, Romero R, Yeo L, Gomez-Lopez N, Chaemsaithong P, Jaovisidha A, et al. The etiology of preeclampsia. Am J Obstet Gynecol. 2022;226(2):S844–S866. doi: 10.1016/j.ajog.2021.11.1356 35177222 PMC8988238

[16] Wilson SL, Leavey K, Cox BJ, Robinson WP. Mining DNA methylation alterations towards a classification of placental pathologies. Hum Mol Genet. 2018;27(1):135–146. doi: 10.1093/hmg/ddx391 29092053 PMC5886226

[17] Kornacki J, Olejniczak O, Sibiak R, Gutaj P, Wender-Ożegowska E. Pathophysiology of Pre-Eclampsiaâ€”Two Theories of the Development of the Disease. Int J Mol Sci. 2023;25(1):307. doi: 10.3390/ijms25010307 38203478 PMC10779413

[18] Roberts JM, Hubel CA. The two stage model of preeclampsia: variations on the theme. Placenta. 2009;30:32–37. doi: 10.1016/j.placenta.2008.11.009 19070896 PMC2680383

[19] Turbeville HR, Sasser JM. Preeclampsia beyond pregnancy: long-term consequences for mother and child. Am J Physiol Renal Physiol. 2020;318(6):F1315–F1326. doi: 10.1152/ajprenal.00071.2020 32249616 PMC7311709

[20] Kingdom J, Ashwal E, Lausman A, Liauw J, Soliman N, Figueiro-Filho E, et al. Guideline no. 442: fetal growth restriction: screening, diagnosis, and management in singleton pregnancies. J Obstet Gynaecol Can. 2023;45(10):102154. doi: 10.1016/j.jogc.2023.05.022 37730302

[21] Burton GJ, Jauniaux E. Pathophysiology of placental-derived fetal growth restriction. Am J Obstet Gynecol. 2018;218(2):S745–S761. 29422210 10.1016/j.ajog.2017.11.577

[22] Colella M, Frérot A, Novais AR, Baud O. Neonatal and long-term consequences of fetal growth restriction. Curr Pediatr Rev. 2018;14(4):212–218. doi: 10.2174/1573396314666180712114531 29998808 PMC6416241

[23] Knöfler M, Haider S, Saleh L, Pollheimer J, Gamage TK, James J. Human placenta and trophoblast development: key molecular mechanisms and model systems. Cell Mol Life Sci. 2019;76:3479–3496. doi: 10.1007/s00018-019-03104-6 31049600 PMC6697717

[24] Hod T, Cerdeira AS, Karumanchi SA. Molecular mechanisms of preeclampsia. Cold Spring Harb Perspect Med. 2015;5(10):a023473. doi: 10.1101/cshperspect.a023473 26292986 PMC4588136

[25] Yeung KR, Chiu CL, Pidsley R, Makris A, Hennessy A, Lind JM. DNA methylation profiles in preeclampsia and healthy control placentas. Am J Physiol Heart Circ Physiol. 2016;310(10):H1295–H1303. doi: 10.1152/ajpheart.00958.2015 26968548

[26] Alberry M, Maddocks D, Jones M, Abdel Hadi M, Abdel-Fattah S, Avent N, et al. Free fetal DNA in maternal plasma in anembryonic pregnancies: confirmation that the origin is the trophoblast. Prenat Diagn. 2007;27(5):415–418. doi: 10.1002/pd.1700 17286310

[27] Guibert J, Benachi A, Grebille AG, Ernault P, Zorn JR, Costa JM. Kinetics of SRY gene appearance in maternal serum: detection by real time PCR in early pregnancy after assisted reproductive technique. Hum Reprod. 2003;18(8):1733–1736. doi: 10.1093/humrep/deg320 12871892

[28] Tjoa ML, Cindrova-Davies T, Spasic-Boskovic O, Bianchi DW, Burton GJ. Trophoblastic oxidative stress and the release of cell-free feto-placental DNA. Am J Pathol. 2006;169(2):400–404. doi: 10.2353/ajpath.2006.060161 16877342 PMC1698796

[29] Phillippe M, Adeli S. Cell-free DNA release by mouse placental explants. PLoS ONE. 2017;12(6):e0178845. doi: 10.1371/journal.pone.0178845 28622381 PMC5473530

[30] Van Boeckel SR, Macpherson H, Norman JE, Davidson DJ, Stock SJ. Inflammation-mediated generation and inflammatory potential of human placental cell-free fetal DNA. Placenta. 2020;93:49–55. doi: 10.1016/j.placenta.2020.02.016 32250739 PMC7146537

[31] Kazemi NY, Fedyshyn B, Yelsa I, Fedyshyn Y, Ruano R, Markovic SN, et al. Increased cell-free fetal DNA release after apoptosis and sterile inflammation in human trophoblast cells. Am J Reprod Immunol. 2021;86(5):e13483. doi: 10.1111/aji.13483 34233077 PMC8541917

[32] Mhatre M, Adeli S, Norwitz E, Craigo S, Phillippe M, Edlow A. The effect of maternal obesity on placental cell-free DNA release in a mouse model. Reprod Sci. 2019;26(9):1218–1224. doi: 10.1177/1933719118811647 30453834 PMC6794655

[33] Flori E, Doray B, Gautier E, Kohler M, Ernault P, Flori J, et al. Circulating cell-free fetal DNA in maternal serum appears to originate from cyto-and syncytio-trophoblastic cells. Case Reports Hum Reprod. 2004;19(3):723–724. 14998976 10.1093/humrep/deh117

[34] Masuzaki H, Miura K, Yoshiura K, Yoshimura S, Niikawa N, Ishimaru T. Detection of cell free placental DNA in maternal plasma: direct evidence from three cases of confined placental mosaicism. J Med Genet. 2004;41(4):289–292. doi: 10.1136/jmg.2003.015784 15060106 PMC1735725

[35] Mao J, Wang T, Wang BJ, Liu YH, Li H, Zhang J, et al. Confined placental origin of the circulating cell free fetal DNA revealed by a discordant non-invasive prenatal test result in a trisomy 18 pregnancy. Clin Chim Acta. 2014;433:190–193. doi: 10.1016/j.cca.2014.03.011 24667696

[36] Hochstenbach R, Nikkels PG, Elferink MG, Oudijk MA, van Oppen C, van Zon P, et al. Cell-free fetal DNA in the maternal circulation originates from the cytotrophoblast: proof from an unique case. Clinical Case Reports. 2015;3(6):489. doi: 10.1002/ccr3.285 26185654 PMC4498868

[37] Van Opstal D, van Veen S, Joosten M, Diderich KE, Govaerts LC, Polak J, et al. Placental studies elucidate discrepancies between NIPT showing a structural chromosome aberration and a differently abnormal fetal karyotype. Prenat Diagn. 2019;39(11):1016–1025. doi: 10.1002/pd.5531 31321790 PMC6899775

[38] Bonanni G, Trevisan V, Zollino M, De Santis M, Romanzi F, Lanzone A, et al. Case Report: Challenges of Non-Invasive Prenatal Testing (NIPT): A Case Report of Confined Placental Mosaicism and Clinical Considerations. Front Genet. 2022;13:881284. doi: 10.3389/fgene.2022.881284 35646091 PMC9134238

[39] Zhao Q, Chen J, Ren L, Zhang H, Liu D, Xi X, et al. Two cases of placental trisomy 21 mosaicism causing false-negative NIPT results. Mol Cytogenet. 2023;16(1):16. doi: 10.1186/s13039-023-00643-3 37452352 PMC10347865

[40] Faas BH, de Ligt J, Janssen I, Eggink AJ, Wijnberger LD, van Vugt JM, et al. Non-invasive prenatal diagnosis of fetal aneuploidies using massively parallel sequencing-by-ligation and evidence that cell-free fetal DNA in the maternal plasma originates from cytotrophoblastic cells. Expert Opin Biol Ther. 2012;12(sup1):S19–S26. doi: 10.1517/14712598.2012.670632 22500971

[41] Chim SS, Tong YK, Chiu RW, Lau TK, Leung TN, Chan LY, et al. Detection of the placental epigenetic signature of the maspin gene in maternal plasma. Proc Natl Acad Sci U S A. 2005;102(41):14753–14758. doi: 10.1073/pnas.0503335102 16203989 PMC1253547

[42] Chan KA, Ding C, Gerovassili A, Yeung SW, Chiu RW, Leung TN, et al. Hypermethylated RASSF1A in maternal plasma: a universal fetal DNA marker that improves the reliability of noninvasive prenatal diagnosis. Clin Chem. 2006;52(12):2211–2218. doi: 10.1373/clinchem.2006.074997 17068167

[43] Tong YK, Jin S, Chiu RW, Ding C, Chan KA, Leung TY, et al. Noninvasive prenatal detection of trisomy 21 by an epigenetic–genetic chromosome-dosage approach. Clin Chem. 2010;56(1):90–98. doi: 10.1373/clinchem.2009.134114 19850629

[44] Lim JH, Lee DE, Kim KS, Kim HJ, Lee BY, Park SY, et al. Non-invasive detection of fetal trisomy 21 using fetal epigenetic biomarkers with a high CpG density. Clin Chem Lab Med. 2014;52(5):641–647. doi: 10.1515/cclm-2013-0802 24353143

[45] Lun FM, Chiu RW, Sun K, Leung TY, Jiang P, Chan KA, et al. Noninvasive prenatal methylomic analysis by genomewide bisulfite sequencing of maternal plasma DNA. Clin Chem. 2013;59(11):1583–1594. doi: 10.1373/clinchem.2013.212274 23857673

[46] Jensen TJ, Kim SK, Zhu Z, Chin C, Gebhard C, Lu T, et al. Whole genome bisulfite sequencing of cell-free DNA and its cellular contributors uncovers placenta hypomethylated domains. Genome Biol. 2015;16:1–11. 25886572 10.1186/s13059-015-0645-x PMC4427941

[47] Lo YD, Leung TN, Tein MS, Sargent IL, Zhang J, Lau TK, et al. Quantitative abnormalities of fetal DNA in maternal serum in preeclampsia. Clin Chem. 1999;45(2):184–188. 9931039

[48] Sekizawa A, Jimbo M, Saito H, Iwasaki M, Matsuoka R, Okai T, et al. Cell-free fetal DNA in the plasma of pregnant women with severe fetal growth restriction. Am J Obstet Gynecol. 2003;188(2):480–484. doi: 10.1067/mob.2003.27 12592259

[49] Zhong XY, Laivuori H, Livingston JC, Ylikorkala O, Sibai BM, Holzgreve W, et al. Elevation of both maternal and fetal extracellular circulating deoxyribonucleic acid concentrations in the plasma of pregnant women with preeclampsia. Am J Obstet Gynecol. 2001;184(3):414–419. doi: 10.1067/mob.2001.109594 11228496

[50] Sekizawa A, Jimbo M, Saito H, Iwasaki M, Sugito Y, Yukimoto Y, et al. Increased cell-free fetal DNA in plasma of two women with invasive placenta. Clin Chem. 2002;48(2):353–354. 11805017

[51] Wataganara T, LeShane ES, Chen AY, Borgatta L, Peter I, Johnson KL, et al. Plasma *γ*-globin gene expression suggests that fetal hematopoietic cells contribute to the pool of circulating cell-free fetal nucleic acids during pregnancy. Clin Chem. 2004;50(4):689–693. 15044329 10.1373/clinchem.2003.030064

[52] Koh W, Pan W, Gawad C, Fan HC, Kerchner GA, Wyss-Coray T, et al. Noninvasive in vivo monitoring of tissue-specific global gene expression in humans. Proc Natl Acad Sci U S A. 2014;111(20):7361–7366. doi: 10.1073/pnas.1405528111 24799715 PMC4034220

[53] Ng EK, Tsui NB, Lau TK, Leung TN, Chiu RW, Panesar NS, et al. mRNA of placental origin is readily detectable in maternal plasma. Proc Natl Acad Sci U S A. 2003;100(8):4748–4753. doi: 10.1073/pnas.0637450100 12644709 PMC153627

[54] Lo YD, Tein MS, Lau TK, Haines CJ, Leung TN, Poon PM, et al. Quantitative analysis of fetal DNA in maternal plasma and serum: implications for noninvasive prenatal diagnosis. Am J Hum Genet. 1998;62(4):768–775. doi: 10.1086/301800 9529358 PMC1377040

[55] Ariga H, Ohto H, Busch MP, Imamura S, Watson R, Reed W, et al. Kinetics of fetal cellular and cell-free DNA in the maternal circulation during and after pregnancy: implications for noninvasive prenatal diagnosis. Transfusion. 2001;41(12):1524–1530. doi: 10.1046/j.1537-2995.2001.41121524.x 11778067

[56] Zhong XY, Holzgreve W, Hahn S. Cell-free fetal DNA in the maternal circulation does not stem from the transplacental passage of fetal erythroblasts. Mol Hum Reprod. 2002;8(9):864–870. doi: 10.1093/molehr/8.9.864 12200465

[57] Sawyer MR, Adeli S, Phillippe M. Cell-free DNA release by mouse fetal membranes. Reprod Sci. 2019;26:847–857. doi: 10.1177/1933719118817659 30572800

[58] Makrydimas G, Gerovassili A, Sotiriadis A, Kavvadias A, Nicolaides K. Cell-free fetal DNA in celomic fluid. Ultrasound Obstet Gynecol. 2008;32(4):594–595. doi: 10.1002/uog.6117 18663774

[59] Bradshaw JL, Cushen SC, Phillips NR, Goulopoulou S. Circulating cell-free mitochondrial DNA in pregnancy. Physiology. 2022;37(4):187–196. doi: 10.1152/physiol.00037.2021 35001655 PMC9191172

[60] Ma MJL, Yakovenko S, Zhang H, Cheng SH, Apryshko V, Zhavoronkov A, et al. Fetal mitochondrial DNA in maternal plasma in surrogate pregnancies: Detection and topology. Prenat Diagn. 2021;41(3):368–375. doi: 10.1002/pd.5860 33140416 PMC7984455

[61] Sin ST, Jiang P, Deng J, Ji L, Cheng SH, Dutta A, et al. Identification and characterization of extrachromosomal circular DNA in maternal plasma. Proc Natl Acad Sci U S A. 2020;117(3):1658–1665. doi: 10.1073/pnas.1914949117 31900366 PMC6983429

[62] Lo YD, Chan KA, Sun H, Chen EZ, Jiang P, Lun FM, et al. Maternal plasma DNA sequencing reveals the genome-wide genetic and mutational profile of the fetus. Sci Transl Med. 2010;2(61):61ra91–61ra91. doi: 10.1126/scitranslmed.3001720 21148127

[63] Yu SC, Chan KA, Zheng YW, Jiang P, Liao GJ, Sun H, et al. Size-based molecular diagnostics using plasma DNA for noninvasive prenatal testing. Proc Natl Acad Sci U S A. 2014;111(23):8583–8588. doi: 10.1073/pnas.1406103111 24843150 PMC4060699

[64] Nygren AO, Dean J, Jensen TJ, Kruse S, Kwong W, van den Boom D, et al. Quantification of fetal DNA by use of methylation-based DNA discrimination. Clin Chem. 2010;56(10):1627–1635. doi: 10.1373/clinchem.2010.146290 20729299

[65] Chiu RW, Akolekar R, Zheng YW, Leung TY, Sun H, Chan KA, et al. Non-invasive prenatal assessment of trisomy 21 by multiplexed maternal plasma DNA sequencing: large scale validity study. BMJ 2011;342. doi: 10.1136/bmj.c7401 21224326 PMC3019239

[66] van Beek DM, Straver R, Weiss MM, Boon EM, Huijsdens-van Amsterdam K, Oudejans CB, et al. Comparing methods for fetal fraction determination and quality control of NIPT samples. Prenat Diagn. 2017;37(8):769–773. doi: 10.1002/pd.5079 28561435 PMC5599991

[67] Bayindir B, Dehaspe L, Brison N, Brady P, Ardui S, Kammoun M, et al. Noninvasive prenatal testing using a novel analysis pipeline to screen for all autosomal fetal aneuploidies improves pregnancy management. Eur J Hum Genet. 2015;23(10):1286–1293. doi: 10.1038/ejhg.2014.282 25585704 PMC4592078

[68] Xu H, Wang S, Ma LL, Huang S, Liang L, Liu Q, et al. Informative priors on fetal fraction increase power of the noninvasive prenatal screen. Genet Med. 2018;20(8):817–824. doi: 10.1038/gim.2017.186 29120459

[69] Jiang P, Peng X, Su X, Sun K, Yu SC, Chu WI, et al. FetalQuantSD: accurate quantification of fetal DNA fraction by shallow-depth sequencing of maternal plasma DNA. NPJ Genom Med. 2016;1(1):1–7. doi: 10.1038/npjgenmed.2016.13 29263813 PMC5685300

[70] Dang M, Xu H, Zhang J, Wang W, Bai L, Fang N, et al. Inferring fetal fractions from read heterozygosity empowers the noninvasive prenatal screening. Genet Med. 2020;22(2):301–308. doi: 10.1038/s41436-019-0636-5 31467446 PMC7000331

[71] Bai Z, Zhao H, Lin S, Huang L, He Z, Wang H, et al. Evaluation of a microhaplotype-based noninvasive prenatal test in twin gestations: determination of paternity, zygosity, and fetal fraction. Genes. 2020;12(1):26. doi: 10.3390/genes12010026 33375453 PMC7823673

[72] Sparks AB, Struble CA, Wang ET, Song K, Oliphant A. Noninvasive prenatal detection and selective analysis of cell-free DNA obtained from maternal blood: evaluation for trisomy 21 and trisomy 18. Am J Obstet Gynecol. 2012;206(4):319–e1. doi: 10.1016/j.ajog.2012.01.030 22464072

[73] Barrett AN, Xiong L, Tan TZ, Advani HV, Hua R, Laureano-Asibal C, et al. Measurement of fetal fraction in cell-free DNA from maternal plasma using a panel of insertion/deletion polymorphisms. PLoS ONE. 2017;12(10):e0186771. doi: 10.1371/journal.pone.0186771 29084245 PMC5662091

[74] Lim JH, Kim SY, Park SY, Lee SY, Kim MJ, Han YJ, et al. Non-invasive epigenetic detection of fetal trisomy 21 in first trimester maternal plasma. PLoS ONE. 2011;6(11):e27709. doi: 10.1371/journal.pone.0027709 22132128 PMC3223183

[75] Manokhina I, Singh TK, Penaherrera MS, Robinson WP. Quantification of cell-free DNA in normal and complicated pregnancies: overcoming biological and technical issues. PLoS ONE. 2014;9(7):e101500. doi: 10.1371/journal.pone.0101500 24987984 PMC4079713

[76] Blais J, Giroux S, Caron A, Clément V, Rousseau F. Precision of Fetal DNA Fraction Estimation by Quantitative Polymerase Chain Reaction Quantification of a Differently Methylated Target in Noninvasive Prenatal Testing. Lab Med. 2020;51(3):279–287. doi: 10.1093/labmed/lmz068 31755528

[77] Ioannides M, Achilleos A, Kyriakou S, Kypri E, Loizides C, Tsangaras K, et al. Development of a new methylation-based fetal fraction estimation assay using multiplex ddPCR. Mol Genet Genomic Med. 2020;8(2):e1094. doi: 10.1002/mgg3.1094 31821748 PMC7005606

[78] El Khattabi LA, Brun S, Gueguen P, Chatron N, Guichoux E, Schutz S, et al. Performance of semiconductor sequencing platform for non-invasive prenatal genetic screening for fetal aneuploidy: results from a multicenter prospective cohort study in a clinical setting. Ultrasound Obstet Gynecol. 2019;54(2):246–254. doi: 10.1002/uog.20112 30191619

[79] Yu SC, Jiang P, Peng W, Cheng SH, Cheung YT, Tse OO, et al. Single-molecule sequencing reveals a large population of long cell-free DNA molecules in maternal plasma. Proc Natl Acad Sci U S A. 2021;118(50):e2114937118. doi: 10.1073/pnas.2114937118 34873045 PMC8685924

[80] Straver R, Oudejans CB, Sistermans EA, Reinders MJ. Calculating the fetal fraction for noninvasive prenatal testing based on genome-wide nucleosome profiles. Prenat Diagn. 2016;36(7):614–621. doi: 10.1002/pd.4816 26996738 PMC5111749

[81] Kim SK, Hannum G, Geis J, Tynan J, Hogg G, Zhao C, et al. Determination of fetal DNA fraction from the plasma of pregnant women using sequence read counts. Prenat Diagn. 2015;35(8):810–815. doi: 10.1002/pd.4615 25967380

[82] Raman L, Baetens M, De Smet M, Dheedene A, Van Dorpe J, Menten B. PREFACE: In silico pipeline for accurate cell-free fetal DNA fraction prediction. Prenat Diagn. 2019;39(10):925–933. doi: 10.1002/pd.5508 31219182 PMC6771918

[83] Yuan Y, Chai X, Liu N, Gu B, Li S, Gao Y, et al. FF-QuantSC: accurate quantification of fetal fraction by a neural network model. Mol Genet Genomic Med. 2020;8(6):e1232. doi: 10.1002/mgg3.1232 32281746 PMC7284026

[84] Chan KA, Jiang P, Sun K, Cheng YK, Tong YK, Cheng SH, et al. Second generation noninvasive fetal genome analysis reveals de novo mutations, single-base parental inheritance, and preferred DNA ends. Proc Natl Acad Sci U S A. 2016;113(50):E8159–E8168. doi: 10.1073/pnas.1615800113 27799561 PMC5167168

[85] Sun K, Jiang P, Cheng SH, Cheng TH, Wong J, Wong VW, et al. Orientation-aware plasma cell-free DNA fragmentation analysis in open chromatin regions informs tissue of origin. Genome Res. 2019;29(3):418–427. doi: 10.1101/gr.242719.118 30808726 PMC6396422

[86] Sun K, Jiang P, Wong AI, Cheng YK, Cheng SH, Zhang H, et al. Size-tagged preferred ends in maternal plasma DNA shed light on the production mechanism and show utility in noninvasive prenatal testing. Proc Natl Acad Sci U S A. 2018;115(22):E5106–E5114. doi: 10.1073/pnas.1804134115 29760053 PMC5984542

[87] Ah Yin, Cf Peng, Zhao X Caughey BA, Jx Yang, Liu J, et al. Noninvasive detection of fetal subchromosomal abnormalities by semiconductor sequencing of maternal plasma DNA. Proc Natl Acad Sci U S A. 2015;112(47):14670–14675. doi: 10.1073/pnas.1518151112 26554006 PMC4664371

[88] Bedon L, Vuch J, Monego SD, Meroni G, Pecile V, Licastro D. An online tool for fetal fraction prediction based on direct size distribution analysis of maternal cell-free DNA. Biotechniques. 2021;70(2):81–88. doi: 10.2144/btn-2020-0143 33249919

[89] Gazdarica J, Hekel R, Budis J, Kucharik M, Duris F, Radvanszky J, et al. Combination of fetal fraction estimators based on fragment lengths and fragment counts in non-invasive prenatal testing. Int J Mol Sci. 2019;20(16):3959. doi: 10.3390/ijms20163959 31416246 PMC6719007

[90] Jiang P, Sun K, Peng W, Cheng SH, Ni M, Yeung PC, et al. Plasma DNA end-motif profiling as a fragmentomic marker in cancer, pregnancy, and transplantation. Cancer Discov. 2020;10(5):664–673. doi: 10.1158/2159-8290.CD-19-0622 32111602

[91] Kang X, Xia J, Wang Y, Xu H, Jiang H, Xie W, et al. An advanced model to precisely estimate the cell-free fetal DNA concentration in maternal plasma. PLoS ONE. 2016;11(9):e0161928. doi: 10.1371/journal.pone.0161928 27662469 PMC5035032

[92] Oberhofer A, Bronkhorst AJ, Uhlig C, Ungerer V, Holdenrieder S. Tracing the origin of cell-Free DNA molecules through tissue-Specific epigenetic signatures. Diagnostics. 2022;12(8):1834. doi: 10.3390/diagnostics12081834 36010184 PMC9406971

[93] Del Vecchio G, Li Q, Li W, Thamotharan S, Tosevska A, Morselli M, et al. Cell-free DNA methylation and transcriptomic signature prediction of pregnancies with adverse outcomes. Epigenetics. 2021;16(6):642–661. doi: 10.1080/15592294.2020.1816774 33045922 PMC8143248

[94] Tse OO, Jiang P, Cheng SH, Peng W, Shang H, Wong J, et al. Genome-wide detection of cytosine methylation by single molecule real-time sequencing. Proc Natl Acad Sci U S A. 2021;118(5):e2019768118. doi: 10.1073/pnas.2019768118 33495335 PMC7865158

[95] Snyder MW, Kircher M, Hill AJ, Daza RM, Shendure J. Cell-free DNA comprises an in vivo nucleosome footprint that informs its tissues-of-origin. Cell. 2016;164(1):57–68. 26771485 10.1016/j.cell.2015.11.050 PMC4715266

[96] Chan KA, Zhang J, Hui AB, Wong N, Lau TK, Leung TN, et al. Size distributions of maternal and fetal DNA in maternal plasma. Clin Chem. 2004;50(1):88–92. doi: 10.1373/clinchem.2003.024893 14709639

[97] Li Y, Zimmermann B, Rusterholz C, Kang A, Holzgreve W, Hahn S. Size separation of circulatory DNA in maternal plasma permits ready detection of fetal DNA polymorphisms. Clin Chem. 2004;50(6):1002–1011. doi: 10.1373/clinchem.2003.029835 15073090

[98] Chen K, Hu Z, Xia Z, Zhao D, Li W, Tyler JK. The overlooked fact: fundamental need for spike-in control for virtually all genome-wide analyses. Mol Cell Biol. 2016;36(5):662–667. 10.1128/MCB.00970-14 PMC4760223 26711261

[99] Wilson SL, Shen SY, Harmon L, Burgener JM, Triche T, Bratman SV, et al. Sensitive and reproducible cell-free methylome quantification with synthetic spike-in controls. Cell Reports Methods. 2022;2(9). doi: 10.1016/j.crmeth.2022.100294 36160046 PMC9499995

[100] Huppertz B, Kadyrov M, Kingdom JC. Apoptosis and its role in the trophoblast. Am J Obstet Gynecol. 2006;195(1):29–39. doi: 10.1016/j.ajog.2005.07.039 16579915

[101] Rana S, Lemoine E, Granger JP, Karumanchi SA. Preeclampsia: pathophysiology, challenges, and perspectives. Circ Res. 2019;124(7):1094–1112. doi: 10.1161/CIRCRESAHA.118.313276 30920918

[102] Sahai K, Saraswathy S, Yadav TP, Arora D, Krishnan M. Pre-eclampsia: Molecular events to biomarkers. Med J Armed Forces India. 2017;73(2):167–174. doi: 10.1016/j.mjafi.2016.09.001 28924318 PMC5592260

[103] Orozco AF, Jorgez CJ, Horne C, Marquez-Do DA, Chapman MR, Rodgers JR, et al. Membrane protected apoptotic trophoblast microparticles contain nucleic acids: relevance to preeclampsia. Am J Pathol. 2008;173(6):1595–1608. doi: 10.2353/ajpath.2008.080414 18974299 PMC2626372

[104] Gupta AK, Holzgreve W, Huppertz B, Malek A, Schneider H, Hahn S. Detection of fetal DNA and RNA in placenta-derived syncytiotrophoblast microparticles generated in vitro. Clin Chem. 2004;50(11):2187–2190. doi: 10.1373/clinchem.2004.040196 15502097

[105] Bischoff FZ, Lewis DE, Simpson JL. Cell-free fetal DNA in maternal blood: kinetics, source and structure. Hum Reprod Update. 2005;11(1):59–67. doi: 10.1093/humupd/dmh053 15569699

[106] loannou YA, Chen FW. Quantitation of DNA fragmentation in apoptosis. Nucleic Acids Res. 1996;24(5):992–993. doi: 10.1093/nar/24.5.992 8600475 PMC145716

[107] Lokeswara AW, Hiksas R, Irwinda R, Wibowo N. Preeclampsia: from cellular wellness to inappropriate cell death, and the roles of nutrition. Front Cell Dev Biol. 2021;9:726513. doi: 10.3389/fcell.2021.726513 34805141 PMC8602860

[108] Jahr S, Hentze H, Englisch S, Hardt D, Fackelmayer FO, Hesch RD, et al. DNA fragments in the blood plasma of cancer patients: quantitations and evidence for their origin from apoptotic and necrotic cells. Cancer Res. 2001;61(4):1659–1665. 11245480

[109] Yu SC, Choy LL, Lo YD. ‘Longing’ for the Next Generation of Liquid Biopsy: The Diagnostic Potential of Long Cell-Free DNA in Oncology and Prenatal Testing. Mol Diagn Ther. 2023;27(5):563–571. doi: 10.1007/s40291-023-00661-2 37474843 PMC10435595

[110] Kalluri R, LeBleu VS. The biology, function, and biomedical applications of exosomes. Science. 2020;367(6478):eaau6977. doi: 10.1126/science.aau6977 32029601 PMC7717626

[111] Sarker S, Scholz-Romero K, Perez A, Illanes SE, Mitchell MD, Rice GE, et al. Placenta-derived exosomes continuously increase in maternal circulation over the first trimester of pregnancy. J Transl Med. 2014;12:1–19. 25104112 10.1186/1479-5876-12-204 PMC4283151

[112] Salomon C, Torres MJ, Kobayashi M, Scholz-Romero K, Sobrevia L, Dobierzewska A, et al. A gestational profile of placental exosomes in maternal plasma and their effects on endothelial cell migration. PLoS ONE. 2014;9(6):e98667. doi: 10.1371/journal.pone.0098667 24905832 PMC4048215

[113] Sabapatha A, Gercel-Taylor C, Taylor DD. Specific isolation of placenta-derived exosomes from the circulation of pregnant women and their immunoregulatory consequences 1. Am J Reprod Immunol. 2006;56(5–6):345–355. 17076679 10.1111/j.1600-0897.2006.00435.x

[114] Repiská G, Konečná B, Shelke GV, Lässer C, Vlková BI, Minárik G. Is the DNA of placental origin packaged in exosomes isolated from plasma and serum of pregnant women? Clin Chem Lab Med. 2018;56(6):e150–e153. doi: 10.1515/cclm-2017-0560 29306910

[115] Yaşa B, Şahin O, Öcüt E, Seven M, Sözer S. Assessment of fetal rhesus D and gender with cell-free DNA and exosomes from maternal blood. Reprod Sci. 2021;28:562–569. doi: 10.1007/s43032-020-00321-4 32968935

[116] Konečná B, Vlková B, Repiská G, Tóthová L. Transfection of maternal cells with placental extracellular vesicles in preeclampsia. Med Hypotheses. 2020;141:109721. doi: 10.1016/j.mehy.2020.109721 32289644

[117] Jin Y, Chen K, Wang Z, Wang Y, Liu J, Lin L, et al. DNA in serum extracellular vesicles is stable under different storage conditions. BMC Cancer. 2016;16:1–9. doi: 10.1186/s12885-016-2783-2 27662833 PMC5035490

[118] Zhang W, Lu S, Pu D, Zhang H, Yang L, Zeng P, et al. Detection of fetal trisomy and single gene disease by massively parallel sequencing of extracellular vesicle DNA in maternal plasma: a proof-of-concept validation. BMC Med Genomics. 2019;12:1–11. 31684971 10.1186/s12920-019-0590-8 PMC6829814

[119] Phillippe M. Cell-free fetal DNA, telomeres, and the spontaneous onset of parturition. Reprod Sci. 2015;22(10):1186–1201. doi: 10.1177/1933719115592714 26134037

[120] Menon R, Mesiano S, Taylor RN. Programmed fetal membrane senescence and exosome-mediated signaling: a mechanism associated with timing of human parturition. Front Endocrinol. 2017;8:196. doi: 10.3389/fendo.2017.00196 28861041 PMC5562683

[121] Salomon C, Nuzhat Z, Dixon CL, Menon R. Placental exosomes during gestation: liquid biopsies carrying signals for the regulation of human parturition. Curr Pharm Des. 2018;24(9):974–982. doi: 10.2174/1381612824666180125164429 29376493 PMC12302748

[122] Lo YD, Zhang J, Leung TN, Lau TK, Chang AM, Hjelm NM. Rapid clearance of fetal DNA from maternal plasma. Am J Human Genetics. 1999;64(1):218–224. doi: 10.1086/302205 9915961 PMC1377720

[123] Yu SC, Lee SW, Jiang P, Leung TY, Chan KA, Chiu RW, et al. High-resolution profiling of fetal DNA clearance from maternal plasma by massively parallel sequencing. Clin Chem. 2013;59(8):1228–1237. doi: 10.1373/clinchem.2013.203679 23603797

[124] Kustanovich A, Schwartz R, Peretz T, Grinshpun A. Life and death of circulating cell-free DNA. Cancer Biol Ther. 2019;20(8):1057–1067. doi: 10.1080/15384047.2019.1598759 30990132 PMC6606043

[125] Lau TW, Leung TN, Chan LY, Lau TK, Chan KA, Tam WH, et al. Fetal DNA clearance from maternal plasma is impaired in preeclampsia. Clin Chem. 2002;48(12):2141–2146. 12446469

[126] Sin ST, Ji L, Deng J, Jiang P, Cheng SH, Heung MM, et al. Characteristics of fetal extrachromosomal circular DNA in maternal plasma: methylation status and clearance. Clin Chem. 2021;67(5):788–796. doi: 10.1093/clinchem/hvaa326 33615350

[127] Patel K, Nguyen J, Shaha S, Brightwell A, Duan W, Zubkowski A, et al. Loss of polarity regulators initiates gasdermin-E-mediated pyroptosis in syncytiotrophoblasts. Life Science Alliance. 2023;6(10). doi: 10.26508/lsa.202301946 37468163 PMC10355286

[128] Yu SY, Li XL. Pyroptosis and inflammasomes in obstetrical and gynecological diseases. Gynecol Endocrinol. 2021;37(5):385–391. doi: 10.1080/09513590.2021.1871893 33432835

[129] Beharier O, Kajiwara K, Sadovsky Y. Ferroptosis, trophoblast lipotoxic damage, and adverse pregnancy outcome. Placenta. 2021;108:32–38. doi: 10.1016/j.placenta.2021.03.007 33812183 PMC8127417

[130] Yu H, Chen L, Du B. Necroptosis in the pathophysiology of preeclampsia. Cell Cycle. 2023;22(14–16):1713–1725. doi: 10.1080/15384101.2023.2229138 37365800 PMC10446795

[131] Jayashankar SS, Nasaruddin ML, Hassan MF, Dasrilsyah RA, Shafiee MN, Ismail NAS, et al. Non-invasive prenatal testing (NIPT): reliability, challenges, and future directions. Diagnostics. 2023;13(15):2570. doi: 10.3390/diagnostics13152570 37568933 PMC10417786

[132] Chiu RW, Chan KA, Gao Y, Lau VY, Zheng W, Leung TY, et al. Noninvasive prenatal diagnosis of fetal chromosomal aneuploidy by massively parallel genomic sequencing of DNA in maternal plasma. Proc Natl Acad Sci U S A. 2008;105(51):20458–20463. doi: 10.1073/pnas.0810641105 19073917 PMC2600580

[133] Demko Z, Prigmore B, Benn P. A critical evaluation of validation and clinical experience studies in non-invasive prenatal testing for trisomies 21, 18, and 13 and monosomy X. J Clin Med. 2022;11(16):4760. doi: 10.3390/jcm11164760 36012999 PMC9410356

[134] Shear MA, Swanson K, Garg R, Jelin AC, Boscardin J, Norton ME, et al. A systematic review and meta-analysis of cell-free DNA testing for detection of fetal sex chromosome aneuploidy. Prenat Diagn. 2023;43(2):133–143. doi: 10.1002/pd.6298 36588186 PMC10268789

[135] Fiorentino D, et al. Prenatal screening for microdeletions and rare autosomal aneuploidies. Clin Obstet Gynecol. 2023;66(3):579–594. doi: 10.1097/GRF.0000000000000799 37438896

[136] Chen Y, Yu Q, Mao X, Lei W, He M, Lu W. Noninvasive prenatal testing for chromosome aneuploidies and subchromosomal microdeletions/microduplications in a cohort of 42,910 single pregnancies with different clinical features. Hum Genomics. 2019;13:1–8. 31783780 10.1186/s40246-019-0250-2 PMC6884830

[137] Grati FR, Gross SJ. Noninvasive screening by cell-free DNA for 22q11. 2 deletion: benefits, limitations, and challenges. Prenat Diagn. 2019;39(2):70–80. doi: 10.1002/pd.5391 30625249

[138] Hanson B, Scotchman E, Chitty LS, Chandler NJ. Non-invasive prenatal diagnosis (NIPD): how analysis of cell-free DNA in maternal plasma has changed prenatal diagnosis for monogenic disorders. Clin Sci. 2022;136(22):1615–1629. doi: 10.1042/CS20210380 36383187 PMC9670272

[139] Zhong LPW, Chiu RW. The next frontier in noninvasive prenatal diagnostics: cell-free fetal DNA analysis for monogenic disease assessment. Annu Rev Genomics Hum Genet. 2022;23(1):413–425. doi: 10.1146/annurev-genom-110821-113411 35316613

[140] Fan HC, Gu W, Wang J, Blumenfeld YJ, El-Sayed YY, Quake SR. Non-invasive prenatal measurement of the fetal genome. Nature. 2012;487(7407):320–324. doi: 10.1038/nature11251 22763444 PMC3561905

[141] Kitzman JO, Snyder MW, Ventura M, Lewis AP, Qiu R, Simmons LE, et al. Noninvasive whole-genome sequencing of a human fetus. Sci Transl Med. 2012;4(137):137ra76–137ra76. doi: 10.1126/scitranslmed.3004323 22674554 PMC3379884

[142] Vong JS, Jiang P, Cheng SH, Lee WS, Tsang JC, Leung TY, et al. Enrichment of fetal and maternal long cell-free DNA fragments from maternal plasma following DNA repair. Prenat Diagn. 2019;39(2):88–99. doi: 10.1002/pd.5406 30575063 PMC6619283

[143] Del Gobbo GF, Yin Y, Choufani S, Butcher EA, Wei J, Rajcan-Separovic E, et al. Genomic imbalances in the placenta are associated with poor fetal growth. Mol Med. 2021;27:1–12. 33413077 10.1186/s10020-020-00253-4 PMC7792164

[144] Lokk K, Modhukur V, Rajashekar B, Märtens K, Mägi R, Kolde R, et al. DNA methylome profiling of human tissues identifies global and tissue-specific methylation patterns. Genome Biol. 2014;15:1–14. doi: 10.1186/gb-2014-15-4-r54 24690455 PMC4053947

[145] Jin Z, Liu Y. DNA methylation in human diseases. Genes Dis. 2018;5(1):1–8. doi: 10.1016/j.gendis.2018.01.002 30258928 PMC6147084

[146] Bergman Y, Cedar H. DNA methylation dynamics in health and disease. Nat Struct Mol Biol. 2013;20(3):274–281. doi: 10.1038/nsmb.2518 23463312

[147] Pastor WA, Kwon SY. Distinctive aspects of the placental epigenome and theories as to how they arise. Cell Mol Life Sci. 2022;79(11):569. doi: 10.1007/s00018-022-04568-9 36287261 PMC9606139

[148] Chu T, Shaw P, McClain L, Simhan H, Peters D. High-resolution epigenomic liquid biopsy for noninvasive phenotyping in pregnancy. Prenat Diagn. 2021;41(1):61–69. doi: 10.1002/pd.5833 33002217

[149] Li S, Zeng W, Ni X, Liu Q, Li W, Stackpole ML, et al. Comprehensive tissue deconvolution of cell-free DNA by deep learning for disease diagnosis and monitoring. Proc Natl Acad Sci U S A. 2023;120(28):e2305236120. doi: 10.1073/pnas.2305236120 37399400 PMC10334733

[150] Lui YY, Chik KW, Chiu RW, Ho CY, Lam CW, Lo YD. Predominant hematopoietic origin of cell-free DNA in plasma and serum after sex-mismatched bone marrow transplantation. Clin Chem. 2002;48(3):421–427. 11861434

[151] Poon LL, Leung TN, Lau TK, Chow KC, Lo YD. Differential DNA methylation between fetus and mother as a strategy for detecting fetal DNA in maternal plasma. Clin Chem. 2002;48(1):35–41. 11751536

[152] Chim SS, Jin S, Lee TY, Lun FM, Lee WS, Chan LY, et al. Systematic search for placental DNA-methylation markers on chromosome 21: toward a maternal plasma-based epigenetic test for fetal trisomy 21. Clin Chem. 2008;54(3):500–511. doi: 10.1373/clinchem.2007.098731 18202156

[153] Yamada Y, Watanabe H, Miura F, Soejima H, Uchiyama M, Iwasaka T, et al. A comprehensive analysis of allelic methylation status of CpG islands on human chromosome 21q. Genome Res. 2004;14(2):247–266. doi: 10.1101/gr.1351604 14762061 PMC327100

[154] Rahat B, Thakur S, Bagga R, Kaur J. Epigenetic regulation of STAT5A and its role as fetal DNA epigenetic marker during placental development and dysfunction. Placenta. 2016;44:46–53. doi: 10.1016/j.placenta.2016.06.003 27452437

[155] Kim HJ, Kim SY, Lim JH, Kwak DW, Park SY, Ryu HM. Quantification and application of potential epigenetic markers in maternal plasma of pregnancies with hypertensive disorders. Int J Mol Sci. 2015;16(12):29875–29888. doi: 10.3390/ijms161226201 26694356 PMC4691144

[156] Chen X, Xiong L, Zeng T, Xiao K, Huang Y, Guo H, et al. Hypermethylated ERG as a cell-free fetal DNA biomarker for non-invasive prenatal testing of Down syndrome. Clin Chim Acta. 2015;444:289–292. doi: 10.1016/j.cca.2015.02.044 25749407

[157] Tsui DW, Lam YD, Lee WS, Leung TY, Lau TK, Lau ET, et al. Systematic identification of placental epigenetic signatures for the noninvasive prenatal detection of Edwards syndrome. PLoS ONE. 2010;5(11):e15069. doi: 10.1371/journal.pone.0015069 21152411 PMC2994810

[158] Lim JH, Kim MH, Park SY, Ryu HM, et al. Novel epigenetic markers on chromosome 21 for noninvasive prenatal testing of fetal trisomy 21. J Mol Diagn. 2016;18(3):378–387. doi: 10.1016/j.jmoldx.2015.12.002 26947512

[159] Lim JH, Lee DE, Park SY, Kim DJ, Ahn HK, Han YJ, et al. Disease specific characteristics of fetal epigenetic markers for non-invasive prenatal testing of trisomy 21. BMC Med Genomics. 2014;7:1–11. doi: 10.1186/1755-8794-7-1 24397966 PMC3892082

[160] Della Ragione F, Mastrovito P, Campanile C, Conti A, Papageorgiou EA, Hultén MA, et al. Differential DNA methylation as a tool for noninvasive prenatal diagnosis (NIPD) of X chromosome aneuploidies. J Mol Diagn. 2010;12(6):797–807. doi: 10.2353/jmoldx.2010.090199 20847278 PMC2963918

[161] Cirkovic A, Garovic V, Milin Lazovic J, Milicevic O, Savic M, Rajovic N, et al. Systematic review supports the role of DNA methylation in the pathophysiology of preeclampsia: a call for analytical and methodological standardization. Biol Sex Differ. 2020;11:1–17. 32631423 10.1186/s13293-020-00313-8 PMC7336649

[162] Lee S, Kim YN, Im D, Cho SH, Kim J, Kim JH, et al. DNA Methylation and gene expression patterns are widely altered in fetal growth restriction and associated with FGR development. Animal Cells Syst. 2021;25(3):128–135. doi: 10.1080/19768354.2021.1925741 34262655 PMC8253195

[163] De Borre M, Che H, Yu Q, Lannoo L, De Ridder K, Vancoillie L, et al. Cell-free DNA methylome analysis for early preeclampsia prediction. Nat Med. 2023;29(9):2206–2215. doi: 10.1038/s41591-023-02510-5 37640858

[164] Gordevičius J, Narmontė M, Gibas P, Kvederavičiūtė K, Tomkutė V, Paluoja P, et al. Identification of fetal unmodified and 5-hydroxymethylated CG sites in maternal cell-free DNA for non-invasive prenatal testing. Clin Epigenetics. 2020;12:1–14. doi: 10.1186/s13148-020-00938-x 33081811 PMC7574562

[165] Holdenrieder S, Stieber P, Chan LY, Geiger S, Kremer A, Nagel D, et al. Cell-free DNA in serum and plasma: comparison of ELISA and quantitative PCR. Clin Chem. 2005;51(8):1544–1546. doi: 10.1373/clinchem.2005.049320 16040855

[166] McAnena P, Brown JA, Kerin MJ. Circulating nucleosomes and nucleosome modifications as biomarkers in cancer. Cancer. 2017;9(1):5. doi: 10.3390/cancers9010005 28075351 PMC5295776

[167] Verhoeven JG, Baan CC, Peeters AM, Clahsen-van Groningen MC, Nieboer D, Herzog M, et al. Circulating cell-free nucleosomes as biomarker for kidney transplant rejection: a pilot study. Clin Epigenetics. 2021;13:1–8. 33573704 10.1186/s13148-020-00969-4 PMC7879674

[168] Guiot J, Struman I, Chavez V, Henket M, Herzog M, Scoubeau K, et al. Altered epigenetic features in circulating nucleosomes in idiopathic pulmonary fibrosis. Clin Epigenetics. 2017;9:1–7. 28824731 10.1186/s13148-017-0383-x PMC5558769

[169] Zhang B, Kim MY, Elliot G, Zhou Y, Zhao G, Li D, et al. Human placental cytotrophoblast epigenome dynamics over gestation and alterations in placental disease. Dev Cell. 2021;56(9):1238–1252. doi: 10.1016/j.devcel.2021.04.001 33891899 PMC8650129

[170] Xu Y, Kang X, Jiang H, Liu H, Wang W. HDAC4 regulates the proliferation, migration, and invasion of trophoblasts in pre-eclampsia through the miR-134-5p/FOXM1 axis. Mol Reprod Dev. 2023;90(12):849–860. doi: 10.1002/mrd.23706 37769062

[171] Meister S, Kolben T, Beyer S, Hutter S, Hofmann S, Kuhn C, et al. Sex-specific epigenetic gene activation profiles are differentially modulated in human placentas affected by intrauterine growth restriction. J Reprod Immunol. 2020;139:103124. doi: 10.1016/j.jri.2020.103124 32289580

[172] Hepp P, Hutter S, Knabl J, Hofmann S, Kuhn C, Mahner S, et al. Histone H3 lysine 9 acetylation is downregulated in GDM Placentas and Calcitriol supplementation enhanced this effect. Int J Mol Sci. 2018;19(12):4061. doi: 10.3390/ijms19124061 30558244 PMC6321349

[173] Meister S, Kellner I, Beyer S, Corradini S, Schulz C, Rogenhofer N, et al. Epigenetic changes occur in placentas of spontaneous and recurrent miscarriages. J Reprod Immunol. 2022;149:103466. doi: 10.1016/j.jri.2021.103466 34929495

[174] Bouvier S, Mousty E, Fortier M, Demattei C, Mercier E, Nouvellon E, et al. Placenta-mediated complications: Nucleosomes and free DNA concentrations differ depending on subtypes. J Thromb Haemost. 2020;18(12):3371–3380. doi: 10.1111/jth.15105 32979032

[175] Fan HC, Blumenfeld YJ, Chitkara U, Hudgins L, Quake SR. Analysis of the size distributions of fetal and maternal cell-free DNA by paired-end sequencing. Clin Chem. 2010;56(8):1279–1286. doi: 10.1373/clinchem.2010.144188 20558635

[176] Ivanov M, Baranova A, Butler T, Spellman P, Mileyko V. Non-random fragmentation patterns in circulating cell-free DNA reflect epigenetic regulation. BMC Genomics. 2015;16:1–12. 26693644 10.1186/1471-2164-16-S13-S1 PMC4686799

[177] Jiang P, Tong YK, Sun K, Cheng SH, Leung TY, Chan KA, et al. Gestational age assessment by methylation and size profiling of maternal plasma DNA: a feasibility study. Clin Chem. 2017;63(2):606–608. doi: 10.1373/clinchem.2016.265702 27979959

[178] An Y, Zhao X, Zhang Z, Xia Z, Yang M, Ma L, et al. DNA methylation analysis explores the molecular basis of plasma cell-free DNA fragmentation. Nat Commun. 2023;14(1):287. doi: 10.1038/s41467-023-35959-6 36653380 PMC9849216

[179] Novakovic B, Yuen RK, Gordon L, Penaherrera MS, Sharkey A, Moffett A, et al. Evidence for widespread changes in promoter methylation profile in human placenta in response to increasing gestational age and environmental/stochastic factors. BMC Genomics. 2011;12:1–14. 10.1186/1471-2164-12-529 PMC3216976 22032438

[180] Gai W, Yu SC, Chan WC, Peng W, Lau SL, Leung TY, et al. Droplet digital PCR is a cost-effective method for analyzing long cell-free DNA in maternal plasma: Application in preeclampsia. Prenat Diagn. 2023;43(11):1385–1393. doi: 10.1002/pd.6432 37655424

[181] Gekas J, Boomer TH, Rodrigue MA, Jinnett KN, Bhatt S. Use of cell-free signals as biomarkers for early and easy prediction of preeclampsia. Front Med. 2023;10:1191163. doi: 10.3389/fmed.2023.1191163 37293304 PMC10244626

[182] Qiao L, Yu B, Liang Y, Zhang C, Wu X, Xue Y, et al. Sequencing shorter cfDNA fragments improves the fetal DNA fraction in noninvasive prenatal testing. Am J Obstet Gynecol. 2019;221(4):345–e1. 10.1016/j.ajog.2019.05.023 31125545

[183] Zhou Z, Ma MJL, Chan RW, Lam WJ, Peng W, Gai W, et al. Fragmentation landscape of cell-free DNA revealed by deconvolutional analysis of end motifs. Proc Natl Acad Sci U S A. 2023;120(17):e2220982120. doi: 10.1073/pnas.2220982120 37075072 PMC10151549

[184] Han DS, Ni M, Chan RW, Chan VW, Lui KO, Chiu RW, et al. The biology of cell-free DNA fragmentation and the roles of DNASE1, DNASE1L3, and DFFB. Am J Hum Genet. 2020;106(2):202–214. doi: 10.1016/j.ajhg.2020.01.008 32004449 PMC7010979

[185] Serpas L, Chan RW, Jiang P, Ni M, Sun K, Rashidfarrokhi A, et al. Dnase1l3 deletion causes aberrations in length and end-motif frequencies in plasma DNA. Proc Natl Acad Sci U S A. 2019;116(2):641–649. doi: 10.1073/pnas.1815031116 30593563 PMC6329986

[186] Chandrananda D, Thorne NP, Bahlo M. High-resolution characterization of sequence signatures due to non-random cleavage of cell-free DNA. BMC Med Genomics. 2015;8:1–19. 26081108 10.1186/s12920-015-0107-z PMC4469119

[187] Han BW, Yang F, Guo ZW, Ouyang GJ, Liang ZK, Weng RT, et al. Noninvasive inferring expressed genes and in vivo monitoring of the physiology and pathology of pregnancy using cell-free DNA. Am J Obstet Gynecol. 2021;224(3):300–e1. 10.1016/j.ajog.2020.08.104 32871130

[188] Ulz P, Thallinger GG, Auer M, Graf R, Kashofer K, Jahn SW, et al. Inferring expressed genes by whole-genome sequencing of plasma DNA. Nat Genet. 2016;48(10):1273–1278. doi: 10.1038/ng.3648 27571261

[189] Guo Z, Yang F, Zhang J, Zhang Z, Li K, Tian Q, et al. Whole-genome promoter profiling of plasma DNA exhibits diagnostic value for placenta-origin pregnancy complications. Adv Sci. 2020;7(7):1901819. doi: 10.1002/advs.201901819 32274292 PMC7141029

[190] He W, Zhang Y, Wu K, Wang Y, Zhao X, Lv L, et al. Epigenetic phenotype of plasma cell-free DNA in the prediction of early-onset preeclampsia. J Obstet Gynaecol. 2023;43(2):2282100. doi: 10.1080/01443615.2023.2282100 38038254

[191] Xu C, Guo Z, Zhang J, Lu Q, Tian Q, Liu S, et al. Non-invasive prediction of fetal growth restriction by whole-genome promoter profiling of maternal plasma DNA: a nested case–control study. BJOG. 2021;128(2):458–466. doi: 10.1111/1471-0528.16292 32364311 PMC7818264

[192] Zhang M, Li K, Qu S, Guo Z, Wang Y, Yang X, et al. Integrative analyses of maternal plasma cell-free DNA nucleosome footprint differences reveal chromosomal aneuploidy fetuses gene expression profile. J Transl Med. 2022;20(1):536. doi: 10.1186/s12967-022-03735-7 36401256 PMC9673457

[193] Jiang P, Xie T, Ding SC, Zhou Z, Cheng SH, Chan RW, et al. Detection and characterization of jagged ends of double-stranded DNA in plasma. Genome Res. 2020;30(8):1144–1153. doi: 10.1101/gr.261396.120 32801148 PMC7462074

[194] Angert RM, LeShane ES, Yarnell RW, Johnson KL, Bianchi DW. Cell-free fetal DNA in the cerebrospinal fluid of women during the peripartum period. Am J Obstet Gynecol. 2004;190(4):1087–1090. doi: 10.1016/j.ajog.2003.10.562 15118647

[195] Cioni R, Bussani C, Scarselli B, Mello G, Mecacci F, Scarselli G. Detection of fetal DNA in the peritoneal cavity during pregnancy. Eur J Obstet Gynecol Reprod Biol. 2003;107(2):210–211. doi: 10.1016/s0301-2115(02)00262-2 12648872

[196] Koide K, Sekizawa A, Iwasaki M, Matsuoka R, Honma S, Farina A, et al. Fragmentation of cell-free fetal DNA in plasma and urine of pregnant women. Prenat Diagn. 2005;25(7):604–607. doi: 10.1002/pd.1213 16032774

[197] Majer S, Bauer M, Magnet E, Strele A, Giegerl E, Eder M, et al. Maternal urine for prenatal diagnosis—an analysis of cell-free fetal DNA in maternal urine and plasma in the third trimester. Prenat Diagn. 2007;27(13):1219–1223. doi: 10.1002/pd.1875 17968856

[198] Shekhtman EM, Anne K, Melkonyan HS, Robbins DJ, Warsof SL, Umansky SR. Optimization of transrenal DNA analysis: detection of fetal DNA in maternal urine. Clin Chem. 2009;55(4):723–729. doi: 10.1373/clinchem.2008.113050 19181739

[199] Tsui NB, Jiang P, Chow KC, Su X, Leung TY, Sun H, et al. High resolution size analysis of fetal DNA in the urine of pregnant women by paired-end massively parallel sequencing. PLoS ONE. 2012;7(10):e48319. doi: 10.1371/journal.pone.0048319 23118982 PMC3485143

[200] Bianchi DW, LeShane ES, Cowan JM. Large amounts of cell-free fetal DNA are present in amniotic fluid. Clin Chem. 2001;47(10):1867–1869. 11568107

[201] Lapaire O, BIANcHI DW, Peter I, O’Brien B, Stroh H, Cowan JM, et al. Cell-free fetal DNA in amniotic fluid: unique fragmentation signatures in euploid and aneuploid fetuses. Clin Chem. 2007;53(3):405–411. doi: 10.1373/clinchem.2006.076083 17259241

[202] Burnham P, Gomez-Lopez N, Heyang M, Cheng AP, Lenz JS, Dadhania DM, et al. Separating the signal from the noise in metagenomic cell-free DNA sequencing. Microbiome. 2020;8:1–9. 32046792 10.1186/s40168-020-0793-4 PMC7014780

[203] Peter I, Tighiouart H, Lapaire O, Johnson KL, Bianchi DW, Terrin N. Cell-free DNA fragmentation patterns in amniotic fluid identify genetic abnormalities and changes due to storage. Diagn Mol Pathol. 2008;17(3):185–190. doi: 10.1097/PDM.0b013e31815bcdb6 18382362 PMC4459511

[204] Larrabee PB, Johnson KL, Lai C, Ordovas J, Cowan JM, Tantravahi U, et al. Global gene expression analysis of the living human fetus using cell-free messenger RNA in amniotic fluid. JAMA. 2005;293(7):836–842. doi: 10.1001/jama.293.7.836 15713773

[205] Lun FM, Chiu RW, Leung TY, Leung TN, Lau TK, Lo YD. Epigenetic analysis of RASSF1A gene in cell-free DNA in amniotic fluid. Clin Chem. 2007;53(4):796–798. doi: 10.1373/clinchem.2006.084350 17405949

[206] Kyathanahalli C, Snedden M, Hirsch E. Is human labor at term an inflammatory condition? Biol Reprod. 2023;108(1):23–40. 36173900 10.1093/biolre/ioac182 PMC10060716

[207] Banerjee S, Huang Z, Wang Z, Nakashima A, Saito S, Sharma S, et al. Etiological value of sterile inflammation in preeclampsia: is it a non-infectious pregnancy complication? Front Cell Infect Microbiol. 2021;11:694298. doi: 10.3389/fcimb.2021.694298 34485175 PMC8415471

[208] Yeganeh Kazemi N, Fedyshyn B, Sutor S, Fedyshyn Y, Markovic S, Enninga EAL. Maternal monocytes respond to cell-free fetal DNA and initiate key processes of human parturition. J Immunol. 2021;207(10):2433–2444. doi: 10.4049/jimmunol.2100649 34663619 PMC8578468

[209] Leung TN, Zhang J, Lau TK, Hjelm NM, Lo YD. Maternal plasma fetal DNA as a marker for preterm labour. Lancet. 1998;352(9144):1904–1905. doi: 10.1016/S0140-6736(05)60395-9 9863792

[210] Yuwono NL, Warton K, Ford CE. The influence of biological and lifestyle factors on circulating cell-free DNA in blood plasma. Elife. 2021;10:e69679. doi: 10.7554/eLife.69679 34752217 PMC8577835

[211] Fisk N, Atun R. Systematic analysis of research underfunding in maternal and perinatal health. BJOG. 2009;116(3):347–356. doi: 10.1111/j.1471-0528.2008.02027.x 19187366

[212] Rice LW, Cedars MI, Sadovsky Y, Siddiqui NY, Teal SB, Wright JD, et al. Increasing NIH funding for academic departments of obstetrics and gynecology: a call to action. Am J Obstet Gynecol. 2020;223(1):79–e1. doi: 10.1016/j.ajog.2020.03.022 32272090

